# Update on current and new potential immunotherapies in breast cancer, from bench to bedside

**DOI:** 10.3389/fimmu.2024.1287824

**Published:** 2024-02-15

**Authors:** Emmanuelle Alaluf, Michal Mia Shalamov, Amir Sonnenblick

**Affiliations:** ^1^ Medical Oncology Clinic, Institut Jules Bordet, Université Libre de Bruxelles, Brussels, Belgium; ^2^ Department of Immunology, Weizmann Institute of Science, Rehovot, Israel; ^3^ Sackler Faculty of Medicine, Tel Aviv University, Tel Aviv, Israel; ^4^ Oncology Division, Tel Aviv Sourasky Medical Center, Tel Aviv, Israel

**Keywords:** breast cancer, immunotherapies, immunooncology, cancer treament, immune escape mechanisms

## Abstract

Impressive advances have been seen in cancer immunotherapy during the last years. Although breast cancer (BC) has been long considered as non-immunogenic, immunotherapy for the treatment of BC is now emerging as a new promising therapeutic approach with considerable potential. This is supported by a plethora of completed and ongoing preclinical and clinical studies in various types of immunotherapies. However, a significant gap between clinical oncology and basic cancer research impairs the understanding of cancer immunology and immunotherapy, hampering cancer therapy research and development. To exploit the accumulating available data in an optimal way, both fundamental mechanisms at play in BC immunotherapy and its clinical pitfalls must be integrated. Then, clinical trials must be critically designed with appropriate combinations of conventional and immunotherapeutic strategies. While there is room for major improvement, this updated review details the immunotherapeutic tools available to date, from bench to bedside, in the hope that this will lead to rethinking and optimizing standards of care for BC patients.

## Introduction

In the last years, there have been many advances and optimization in the treatment of breast cancer (BC). However, despite such progress, resistance to therapy and disease relapse remain important challenges in the management of BC in a considerable proportion of patients. Specifically, impressive advances have been seen in cancer immunotherapy during the last decade. Cancer immunotherapy exploits the host’s immune system to eradicate tumor cells. Although BC has long been considered a non-immunogenic process, immunotherapy for the treatment of BC is emerging as a new therapeutic approach with considerable potential, supported by a plethora of completed and currently ongoing preclinical and clinical studies in various types of BC immunotherapies. Tumor-infiltrating lymphocytes (TILs) are more commonly found in Human epidermal growth factor receptor 2 (HER-2)-positive BC and triple negative BC (TNBC), where the median percentages are 15% and 20%, respectively (1). However, 10% of TILs are also found in hormone receptor (HR)-positive BC (1). TILs can specifically target tumor cells following activation by antigen presenting cells (APC) via tumor antigen peptide presentation to human leukocyte antigen (HLA) molecules. TILs are associated with a better prognosis in TNBC (2) and node-positive TNBC (1). In HER2-positive BC, the presence of TILs showed contradictory data regarding trastuzumab therapy benefit (3, 4). TILs have also been associated with a higher probability of pathological complete response (pCR) in neoadjuvant settings (5, 6). Likewise, in HR-positive BC, CD8+ T-cell infiltration has been associated with survival (7), although this is currently under debate since contradictory results have been found for this BC subtype in neoadjuvant (8, 9) and adjuvant (10) settings. This led to suggest that the tumor-eradicating properties of TILs are an efficient part of the antitumor immune response and could therefore be exploited as immunotherapy to improve the clinical outcome of BC patients. γδ T-cells and natural killer (NK) cells have also been associated with a better prognosis in all BC subtypes (11, 12). Many targets are constantly being discovered on antitumor lymphoid cells, such as immune checkpoints. In addition, other immune cells of the tumor microenvironment (TME) contribute positively or negatively to the antitumor immune response and are currently a topic of intense preclinical and clinical research, such as tumor-infiltrating myeloid cells (13). Herein, we summarize the current and new potential immunotherapeutic strategies showing promising results in the emerging field of BC immunotherapy. We provide a basis for reflection on the available immunotherapeutic tools to date in the hope that this will lead to rethinking and optimizing standards of care for BC patients.

## Directed monoclonal antibodies

**Figure 1:**
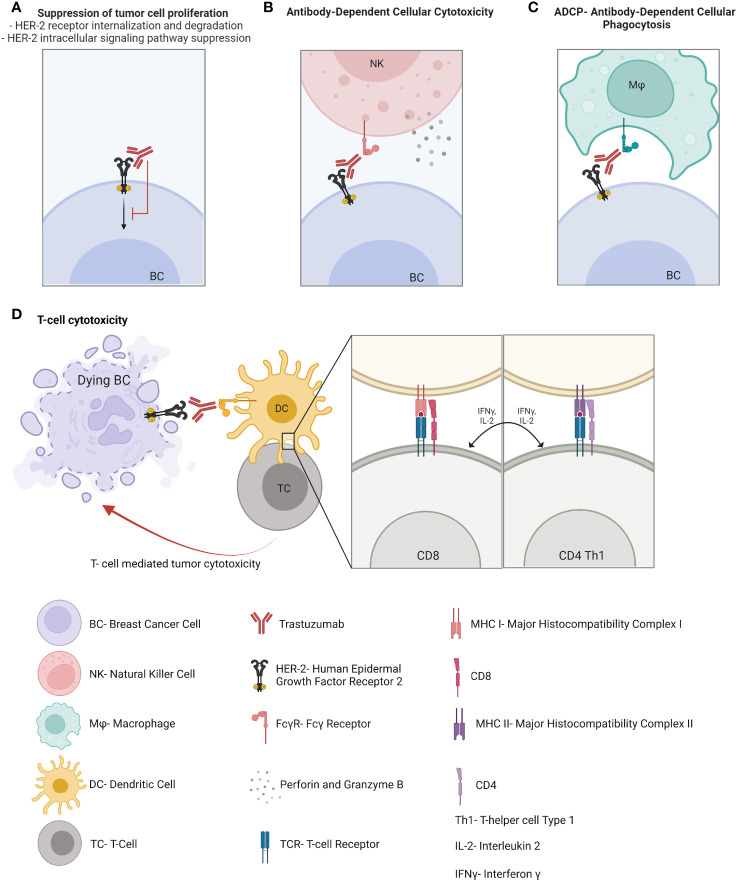
Mechanisms of action of monoclonal antibodies on the antitumor immune response.

HER2 is overexpressed in 15-20% of BC and correlates with higher grade, aggressive phenotype, and poor clinical outcome. Immunotherapies in the form of monoclonal antibodies specifically binding to HER-2 receptor, added to chemotherapy, are the cornerstone for HER-2-overexpressing BC therapy and have led to significant improvements in HER2-positive BC prognosis compared to previous chemotherapy regimens. Trastuzumab has been approved for the treatment of HER2-positive BC patients for approximately the past 20 years and acts through several mechanisms of action. It suppresses the HER2 intracellular signaling pathway by binding to the transmembrane HER2 receptor, which is followed by its internalization, degradation, and downregulation of PI3K pathway. In addition, trastuzumab activates both the innate and adaptive immune systems. Indeed, this monoclonal antibody enhances antibody-dependent cellular cytotoxicity (14), antibody-dependent cellular phagocytosis and macrophage activation (15), Fc-mediated immune priming by dendritic cells (DCs) (16), effector HER-2-specific T cell response (17) and memory T-cell response (16) (**Figure 1**). These mechanisms seem to be critical for the induction of a pCR after neoadjuvant therapy in HER2-positive BC patients (18). Pertuzumab is a dual HER2/HER3 monoclonal antibody approved in combination with trastuzumab and taxane-based chemotherapy for first-line therapy in HER2-positive metastatic BC and in adjuvant and neoadjuvant settings. It works by blocking HER2 heterodimerization and may act by promoting an antitumor immune response (19), although data regarding its mechanisms of action and its synergism with trastuzumab is still limited. To improve the efficacy of trastuzumab, the immunogenic properties of trastuzumab may be exploited in association with other strategies. For example, margetuximab was approved in combination with chemotherapy for third-line therapy in metastatic HER2-positive BC disease. Margetuximab is a monoclonal antibody similar to trastuzumab, whose modified Fc fragment has a much greater affinity for its activating Fcγ receptors and a decreased affinity for its inhibitory Fcγ receptors on tumor-infiltrating NK cells and macrophages. This way margetuximab promotes antibody-dependent cellular cytotoxicity and phagocytosis processes against tumor cells. This may explain the influence of Fcγ receptor polymorphism on overall survival of BC patients treated with margetuximab compared to trastuzumab (20). In light of the success of anti-HER2 therapies in HER2-positive BC, one might wonder why other monoclonal antibodies targeting tumor antigens other than HER2 have not been developed for other BC subtypes. Such monoclonal antibodies, synergizing with the potential antitumoral properties of the TME, might be particularly efficient in BC tumors wherein myeloid cells are abundant.

## Antibody-drug conjugates

**Figure 2:**
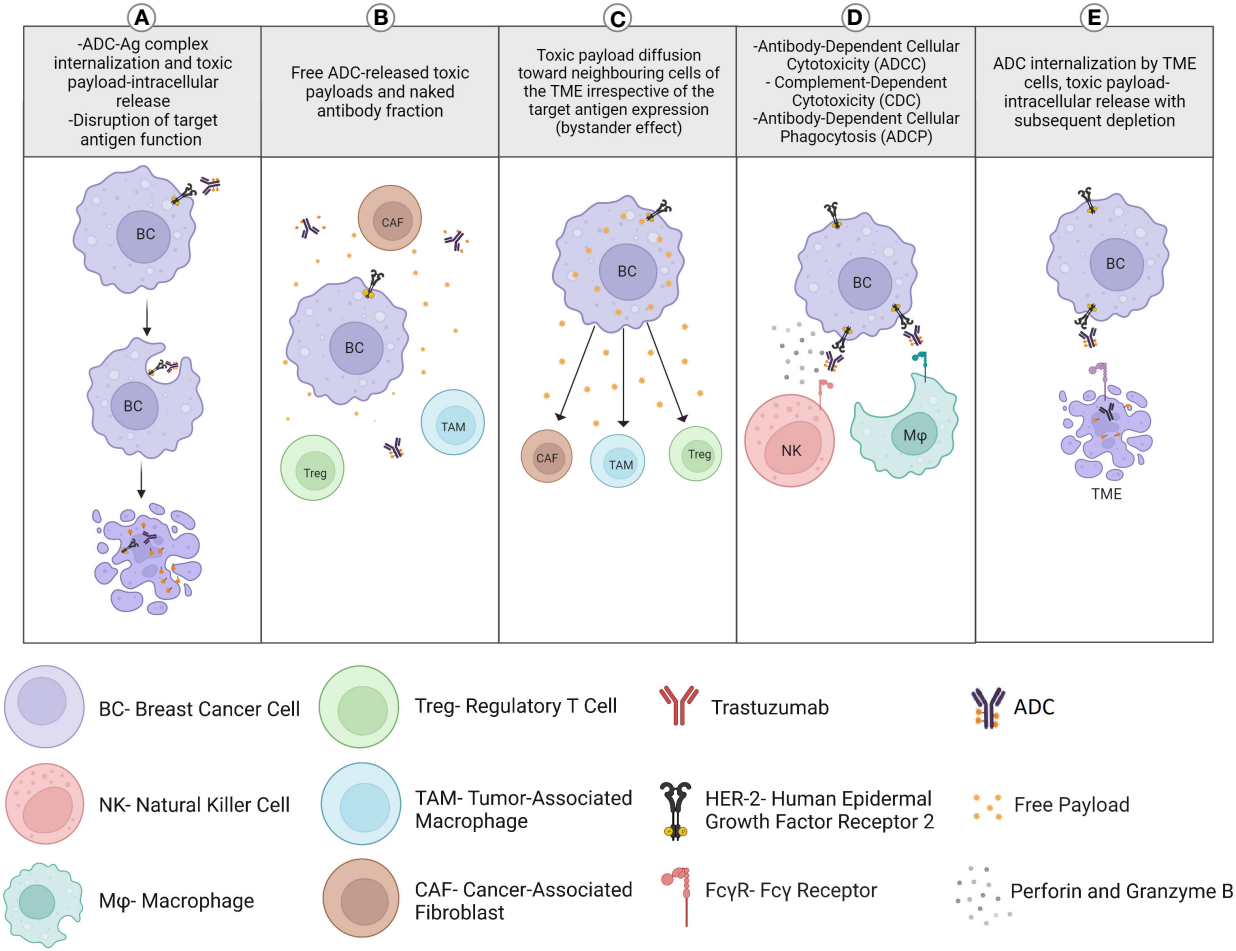
Mechanisms of action of antibody-drug conjugates (ADC) on the antitumor immune response.

Antibody-drug conjugates (ADC), a new emerging class of antineoplastic agents with a high therapeutic index and impressive clinical efficacy, display both immune mechanisms of action, like those of naked directed monoclonal antibodies, combined with the targeted delivery of chemotherapy directly to antigen-expressing tumor cells (21). They are therefore known as “biological missiles”. Recently, other mechanisms of action have been suggested to contribute to both their antitumor activity and adverse events (such as thrombocytopenia). For example, the release of chemotherapy into the TME may lead to the recruitment of particularly immunosuppressive, protumoral and tissue repairing myeloid cells. ADC may be taken up by macrophages through Fcγ receptors, leading to myeloid cell depletion or modulation of the activation state (22). Chemotherapy may also deplete regulatory T cells by diffusing into the TME through a bystander effect. Chemotherapy may also further promote NK cell-mediated antibody-dependent cellular cytotoxicity (ADCC) or regulate tumor antigen presentation by DCs (**Figure 2**). Trastuzumab emtansine is an ADC of trastuzumab covalently linked to the cytotoxic agent emtansine (DM1/maytansinoid). It is approved for HER2-positive BC patients in the metastatic setting and in the adjuvant setting for HER2-positive BC patients with residual invasive disease following neoadjuvant trastuzumab and chemotherapy. Trastuzumab-deruxtecan is the following ADC of trastuzumab linked to the topoisomerase 1 inhibitor deruxtecan (DXt/camptothecin). While TDM-1 has a non-cleavable linker and an average of 3.5 molecules of payloads per antibody, trastuzumab deruxtecan displays a cleavable linker with 8 molecules of a different payload. These differences could affect their antitumor mechanisms of action, such as the bystander effect and cellular toxicity. The efficacy and safety of trastusumab deruxtecan was compared with trastuzumab emtansine in the DESTINY-BREAST03 phase 3 randomized clinical trial, showing a lower risk of disease progression or death (23). Trastuzumab deruxtecan received accelerated approval in 2019 and has now become the new standard of care for second-line therapy in HER2-positive BC patients who have received a prior anti-HER2 based regimen either in the metastatic setting, or in the neoadjuvant or adjuvant setting and have developed disease recurrence during or within 6 months of completing therapy. In addition, it is approved for locally advanced or metastatic HER2-low (IHC 1+ or IHC 2+/FISH‑) BC patients who have received a prior chemotherapy in the metastatic setting or developed disease recurrence during or within six months of completing adjuvant chemotherapy. Moreover, trastuzumab deruxtecan is currently compared, when used with or without pertuzumab, to the standard of care which is taxane, pertuzumab and trastuzumab as first-line treatment in the DESTINY-BREAST09 phase 3 clinical trial. Trastuzumab deruxtecan in association with tucatinib is also currently studied in metastatic BC patients, including with active brain metastasis, in the HER2-CLIMB-04 phase 2 clinical trial. Another ADC, sacituzumab govitecan, targets the human trophoblast cell-surface antigen 2 (Trop-2), which is highly expressed in BC, and is coupled with a high drug-to-antibody ratio to SN-38, the active metabolite of irinotecan. This leads to the delivery of high concentrations of the chemotherapy to the tumor cells by intracellular uptake of SN-38, thereby also allowing the cells of the TME to be eradicated by SN-38 which is released extracellularly from the tumor cells through a bystander effect. This antitumor effect may also be mediated by a significant antibody-dependent cellular cytotoxicity effect against the Trop-2-positive tumor cells (24). Sacituzumab govitecan received accelerated approval in 2020 for the treatment of refractory metastatic TNBC following at least two prior chemotherapies, by showing promising results for this notoriously difficult-to-treat group of patients (25). Recently, it has demonstrated extended progression-free survival (PFS) and overall survival (OS), as well as greater health-related quality of life benefits than chemotherapy, and moved to second-line therapy of TNBC (at least one in the metastatic setting) (25). It may also represent a new option for endocrine-resistant hormone receptor-positive/HER2-negative metastatic BC, since it has recently shown a longer PFS and a statistically significant OS compared to standard chemotherapy (capecitabine, eribulin, vinorelbine or gemcitabine) after CDK4/6 inhibitors and 2 to 4 previous lines of chemotherapy (26). The indication of sacituzumab govitecan is currently investigated in case of residual disease after neoadjuvant chemotherapy.

## Immune checkpoint inhibitors

During the last decade, the emergence of immune checkpoint inhibitors (ICI) has revolutionized the field of cancer therapies, especially in advanced or metastatic cancers where they have shown unprecedented and durable efficacy. They are approved in many different cancer types such as lung cancer, melanoma, renal cell carcinoma, and in any high microsatellite instability or mismatch repair deficiency. Used alone or in combination with other ICI or chemotherapies, they represent a staggering proportion of the ongoing clinical trials in oncology. In all cancer types, the most widely studied immunotherapeutic agents to date are ICI blocking cytotoxic T lymphocyte-associated molecule-4 (CTLA-4), programmed cell death receptor-1 (PD-1) and programmed cell death ligand-1 (PD-L1). While PD-1 is mainly expressed on TILs, PD-L1 is expressed on both cancer cells and tumor-infiltrating immune cells. Although the impact of ICI on the immune response remains to be fully elucidated, the PD-1/PD-L1 immune inhibitory axis is thought to be upregulated in the TME and to impair the effector stage of the antitumor immune response (27).

## PD-1 inhibitors

Nivolumab and pembrolizumab are human monoclonal antibodies that block PD-1 and therefore the interaction of PD-1 with its ligand PD-L1, preventing T-cell suppression.

In TNBC, although pembrolizumab showed antitumor activity in the phase 1b KEYNOTE-012 and the phase 2 KEYNOTE-086 trials, the KEYNOTE-119 phase 3 trial comparing pembrolizumab with chemotherapy did not show significant improvement in OS in the second or third-line treatment of patients with metastatic TNBC (28). However, in the KEYNOTE-355 phase 3 study, pembrolizumab combined with chemotherapy significantly improved PFS compared with chemotherapy alone in patients with advanced or metastatic PD-L1-positive TNBC (CPS≥10) (29). Moreover, the follow-up of the patients with CPS of 10 or more showed a significantly longer OS with no new safety signals identified when pembrolizumab was added to chemotherapy (30). In addition, among patients with previously untreated stage II or III TNBC, the rate of pCR at definitive surgery was higher in pembrolizumab plus neoadjuvant chemotherapy compared with placebo plus chemotherapy in the KEYNOTE-522 phase 3 trial, along with disease-free survival (DFS) (31). Based on these results, the FDA approved in 2021 pembrolizumab for high-risk, early-stage, TNBC, in combination with chemotherapy as neoadjuvant treatment, and then continued as a single agent as adjuvant treatment. The FDA also granted accelerated approval in 2020 to pembrolizumab in combination with chemotherapy for patients with locally advanced or metastatic TNBC whose tumors express PD-L1. Other treatments with the combination of pembrolizumab and eribulin showed promise for patients with metastatic TNBC, with efficacy that seems greater than reports of either drug alone, according to the KEYNOTE-150 phase 1b/2 study (NCT02513472) (32). A pilot study comparing nivolumab with capecitabine and with combination therapy as adjuvant therapy after residual disease following neoadjuvant chemotherapy is under investigation (NCT03487666).

In HER2-positive BC, the combination of pembrolizumab plus trastuzumab demonstrated a tolerable safety profile, activity and durable clinical benefit, in advanced HER-2-positive, trastuzumab-resistant, PD-L1-positive BC disease (33).

In HR-positive BC patients, fewer clinical trials have been performed so far. In a phase 1b study, pembrolizumab was well tolerated with modest but durable partial response in certain patients with previously treated, advanced, PD-L1-positive HR-positive HER2-negative BC (34). In women with early-stage, high-risk, HR-positive HER2-negative BC, an ongoing phase 2 trial in the neoadjuvant setting with pembrolizumab in association with standard chemotherapy showed improved pCR (35). Since CDK4/6 inhibitors induce, in addition to a tumor cell cycle arrest, an enhanced antitumor immune response (36), they may be used in synergy with ICI to increase tumor immunogenicity (NCT02648477). Other studies currently assessing PD-1 inhibitors in different settings are summarized in **Table 1**.

**Table 1:** Published and ongoing clinical trials related to ICI.

TNBC	Setting	Regimens	Results	Safety data	references		
KEYNOTE-119 phase 3	Metastatic	pembro 200mg 1x/3 weeks vs investigator’s choice chemo (capecitabine, eribulin, gemcitabine, or vinorelbine)	OS ≈	Anemia and leupopenia ↘ with pembro vs chemo. AE of grade≥3 in 20% of both groups	(28)		
KEYNOTE-355 phase 3	Metastatic	Chemo (nab-paclitaxel, paclitaxel, or gemcitabine/carboplatin)+pembro 200mg 1x/3 weeks vs placebo	↗ PFS (≥CPS 10) ↗ OS (≥CPS 10): 23 vs 16.1 months	68.1% of AE of grade≥3 in pembro+chemo vs 66.9% in chemo	(29, 30)		
KEYNOTE-522 phase 3	Early (neoadjuvant and adjuvant)	Chemo (paclitaxel+carboplatin)+4 cycles of neoadjuvant pembro 200mg 1x/3 weeks vs placebo. Additional four cycles of pembro vs placebo and then doxorubicin+cyclophosphamide or epirubicin+cyclophosphamide for both groups. Adjuvant pembro vs placebo.	↗ DFS ↗ pCR: 64.8% vs 51.2%	78% of AE of grade≥3 in pembro+chemo vs 73% in chemo	(31)		
KEYNOTE-150 phase 1b/2	Metastatic	Pembro 200mg d1+eribulin 1.4mg/m² d1, d8/21	↗ ORR in PD-L1+ tumors: 28.4% vs 17.3%	26.3% of neutropenia of grade≥3	(32)		
IMpassion130 phase 3	Metastatic	Nab-paclitaxel 100mg/m² d1,8,15/28+atezolizumab 840mg d1, d15 vs placebo	↗ PFS: 7.2 vs 5.5 months in PD-L1+ tumors: 7.5 vs 5 months OS ≈ Withdrawn due to lack of benefit.	48.7% of AE of grade≥3 in the atezo group vs 42.2% in placebo, 57,3% of potential immune -related AE vs 41.8%, respectively	(37, 38)		
IMpassion131 Phase 3	Metastatic	Paclitaxel 90mg/m² d1,8,15/28+atezolizumab 840mg d1 and d15 vs placebo	PFS ≈ OS ≈	53% of AE of grade≥3 in atezo group vs 46% in placebo. Higher incidence of low-grade hypo and hyperthyroidism in atezo group	(39)		
Pilot study	Early (residual disease)	adjuvant capecitabine 1250mg/m² bid d1-d14/21 or nivolumab 360mg 1x/3 weeks or both	Immunoscore change?	?	NCT03487666		
Phase 2	Metastatic	carboplatin+nivolumab 360mg 1x/3 weeks vs placebo	PFS?	?	NCT03414684		
Phase 3	Metastatic	Chemotherapies+pembrolizumab 200mg 1x/3 weeks vs placebo	PFS? OS?	?	NCT02819518		
Phase 1/2	Metastatic	Niraparib up to 300mg/day d1-21+pembrolizumab 200mg d1/21	DLT? ORR?	?	NCT02657889		
IMpassion030 Phase 3	Early (adjuvant)	paclitaxel 80mg/m² 1x/week for 12 weeks followed by dose-dense doxorubicin 60mg/m² or epirubicin 90mg/m²+cyclophosphamide 600mg/m² 1x/2weeks for 4 doses+atezolizumab 840 mg 1x/2 weeks for 10 doses and then maintenance 1200 mg 1x/3weeks to complete 1 year vs placebo	iDFS?	?	NCT03498716		
NeoTRIP Phase 3	Early (neoadjuvant)	Neoadjuvant carboplatin+nab-paclitaxel 125mg/m² d1 and d8+atezolizumab 1200mg 1x/3weeks for 8 cycles vs placebo, followed by adjuvant anthracyclines for 4 cycles	pCR ≈ EFS?	Liver transaminase abnormalities in atezo group	NCT02620280 (40)		
IMpasision031 phase 3	Early (neoadjuvant)	Nab-paclitaxel 125mg/m² 1x/week+atezolizumab 840mg 1x/2weeks for 12 weeks followed by atezolizumab 840mg+doxorubicin 60mg/m²+cyclophosphamide 600mg/m² 1x/2weeks for 4 doses followed by adjuvant atezolizumab 1200mg 1x/3weeks for 11 doses vs placebo	↗ pCR in all-randomized population and PD-L1+ status	30% of AE of grade≥3 in atezo group vs 18% in placebo, such as febrile neutropenia, pneumonia and pyrexia	NCT03197935 (41)		
Phase 3	Early relapsing metastatic	Chemotherapy (capecitabine or gemcitabine/carboplatin) and atezolizumab 1200mg 1x/3weeks vs placebo	OS?	?	NCT03371017		
Phase 3	Early (adjuvant)	Adjuvant avelumab 10mg/kg 1x/2 weeks vs placebo	DFS? OS?	?	NCT02926196		
Phase 2	Stage III (neoadjuvant)	Neoadjuvant ipilimumab 1mg/kg 6 weekly for 2 doses+nivo 240mg every 2 weeks for 6 weeks+weekly paclitaxel 80mg/m² for 12 weeks+postoperative nivolumab 480 mg 4-weekly for further 9 months	pCR 37.5% in PD-L1+ and 23% in PD-L1-, 57.6% of ORR	?	ongoing (42)		
GeparNuevo Phase 2	Early (neoadjuvant)	Nab-paclitaxel 125mg/m² 1x/week+durvalumab 1.5g 1x/4 weeks vs placebo followed by doxorubicin 60mg/m²+cyclophosphamide 600mg/m²+durvalumab vs placebo	pCR ≈ ↗iDFS 85.6% in durva group vs 77.2% in placebo ↗DDFS 91.7% in durva group vs 78.4% in placebo ↗OS 95.2% in durva group vs 83.5% in placebo		NCT02685059		

HER2+ BC	Setting	Regimens	Results	Safety data	references		
PANACEA Phase 1b/2	Metastatic	Trastuzumab 6mg/kg+pembro 200mg 1x/3 weeks vs placebo	OR of 15% in PD-L1+ tumors, 0% in PD-L1- tumors	29% of AE of grade≥3. Immune-related AE in 19% such as thyroid dysfunction, pneumonitis and autoimmune hepatitis.	(33)		
KATE2 Phase 2	Metastatic	TDM-1 3.6mg/kg+atezolizumab 1200mg 1x/3 weeks vs placebo	PFS ≈ In PD-L1+ tumors: PFS 8.5 months in atezo group vs 4.1 months in placebo	19% of AE of grade≥3 in atezo group vs 3% in placebo	(43)		
Phase 2	Early (neoadjuvant)	Neoadjuvant pembro 200mg+trastuzumab 6mg/kg+pertuzumab 420mg/kg 1x/3 weeks	pCR?	?	NCT03988036		
Phase 2	Metastatic	5 administrations of pembro 200mg 1x/3 weeks or VRP-HER2 vaccine 4x10EE8 IU 1x/2 weeks for 3 injections or both of them	Anti-HER2 TILs and antibodies?	?	NCT03632941		
Phase 3	Metastatic	Paclitaxel 1x/week or docetaxel 1x/3 weeks+ trastuzumab+pertuzumab+atezolizumab 1x/3 weeks vs placebo for 2 years	PFS? OS?	?	NCT03199885		
IMpassion050 Phase 3	Early	Neoadjuvant doxorubicin 60mg/m²+cyclophosphamide 600mg/m²+atezolizumab 840 mg 1x/2 weeks vs placebo followed by paclitaxel 80mg/m²1x/week for 12 weeks+trastuzumab 6mg/kg+pertuzumab 420mg 1x/3weeks. Adjuvant to complete up to 1 year HER2-target therapy+atezolizumab 1200mg 1x/3 weeks vs placebo	pCR ≈ DFS? OS?	Neoadjuvant: 47.3% of AE of grade≥3 in atezo group vs 42.2% in placebo Adjuvant: 13.4% of AE of grade≥3 in atezo group vs 9.8% in placebo	NCT03726879 (44)		
Phase 2a	Metastatic	Atezolizumab+paclitaxel+trastuzumab+pertuzumab	Antitumor activity (RECIST)?	?	NCT03125928		
Phase 1b	Metastatic	Durvalumab+trastuzumab 1x/3 weeks	Recommended phase 2 dose?	?	NCT02649686		

Luminal	Setting	Regimens	Results	Safety data	references		
I-SPY2 Phase 2	Early (neoadjuvant)	neoadjuvant paclitaxel 80 mg/m²+pembrolizumab 200mg 1x/3 weeks vs placebo followed by doxorubicin 60 mg/m²+cyclophosphamide 600 mg/m². Standard-of-care adjuvant therapy.	pCR 30% vs 13% for HR+HER2- and 60% vs 22% for TNBC	immune-related AE such as thyroid dysfunction (1,4% ≥3) and adrenal insufficiency (7,2% of ≥3)	NCT01042379 (35)		
Phase 2	Metastatic	Pembrolizumab 1x/3 weeks for 24 months+aromatase inhibitor po QD	ORR? OS?	?	NCT02648477		
Phase 2	Early (residual disease)	Adjuvant pembrolizumab 1x/3 weeks up to 24 months+hormone therapy	DFS? OS	?	NCT02971748		
KEYNOTE-756 phase 3	Early (neoadjuvant)	Neoadjuvant pembrolizumab 200mg 1x/3 weeks vs placebo+paclitaxel 80mg/m² 1x/week followed by doxorubicin 60mg/m² or epirubicin 100mg/m²+cyclophosphamide 600mg/m² 1x/2 weeks. Adjuvant pembrolizumab 200mg 1x/3 weeks vs placebo+hormone therapy	pCR? EFS?	?	NCT03725059		
Phase 3	Metastatic	pembrolizumab 200mg 1x/3 weeks vs placebo+chemotherapy (paclitaxel, nab-paclitaxel, liposomal doxorubicin or capecitabine)	PFS? OS?	?	NCT04895358		
Phase 2	Early (neoadjuvant)	Tremelimumab 3mg/kg+exemestane 25mg daily followed by durvalumab 20mg/kg 1x/4weeks+exemestane 25mg daily	CD8+ T cells? pCR?	?	NCT02997995		
Phase 2	Metastatic	Atezolizumab 840mg 1x/2 weeks+cobimetinib daily	ORR?	?	NCT03566485		
Phase 2b	Metastatic	Chemotherapy (pegylated liposomal doxorubicin 20mg/m² 1x/2 weeks+cyclophosphamide 50mg daily first 2 weeks in each 4 week cycle)+ibilimumab 1mg 1x/6 weeks and nivolumab 240mg 1x/2 weeks vs placebo	PFS?	?	NCT03409198		
Phase 2	Metastatic (hypermutated)	Nivolumab 1x/2 weeks+ibilimumab 1x/6 weeks	ORR?	?	NCT03789110		
Phase 2	Metastatic	sacituzumab govitecan 10mg/kg 2x/3 weeks+pembrolizumab 1x/week vs placebo	PFS? ORR? OS?	?	NCT04448886		
Phase 3	Early	neoadjuvant chemotherapy (paclitaxel followed by anthracycline+cyclophosphamide) and adjuvant hormonotherapy of investigator’s choice+neoadjuvant and adjuvant nivolumab vs placebo	pCR?	?	NCT04109066		

## PD-L1 inhibitors

Atezolizumab, durvalumab and avelumab are human monoclonal antibodies that block PD-L1 and therefore PD-1/PD-L1 interaction, T-cell activation and proliferation.

In TNBC, the association of durvalumab with nab-paclitaxel and doxorubicin-cyclophosphamide neoadjuvant chemotherapy suggested a high rate of pCR in a phase 1/2 trial (43), which was higher in PD-L1-positive and TILs-high than PD-L1-negative patients (44). This has not been observed in the phase 3 trial comparing the addition of atezolizumab to carboplatin and nab-paclitaxel with the chemotherapy regimen alone in a neoadjuvant setting (40). However, the IMpassion130 phase 3 study demonstrated a prolonged PFS with atezolizumab plus nab-paclitaxel in metastatic TNBC patients (37) and a clinically meaningful OS benefit in previously untreated PD-L1-positive patients, compared with placebo plus nab-paclitaxel (38). Following this clinical trial, nab-paclitaxel with atezolizumab received accelerated approval in March 2019 for first-line treatment of locally advanced or metastatic TNBC whose tumors express PD-L1 (>=1%). However, this has been withdrawn after IMpassion131 clinical trial results showing that atezolizumab and paclitaxel under the same settings did not show any improvement of PFS or OS versus paclitaxel alone (39).

The addition of atezolizumab to TDM-1 did not show any improvement and was associated with more adverse events in previously treated HER2-positive locally advanced or metastatic BC patients who received prior trastuzumab and taxane based therapies. However, a benefit in terms of PFS in favor of the combination has been observed in the PD-L1-positive subgroup of patients (45). Further study is required in subpopulations of patients.

Further results from various clinical trials investigating anti-PD-L1 treatments in TNBC, HER2-positive or HR-positive BC patients are summarized in **Table 1** (41, 46).

## CTLA-4 inhibitors

CTLA-4 on TIL surface mediates T-cell suppression by binding to CD80 and CD86 (expressed on the surface of antigen-presenting cells), therefore competing with the co-stimulatory receptor CD28 on the cell surface of T cells (47). Ipilimumab is a human monoclonal antibody that targets CTLA-4, thus preventing its inhibitory effect on T-cell activation. Ipilimumab is under investigation in various settings in BC which are summarized in **Table 1** (42).

## Optimizing trial design

Major clinical trials, such Keynote-119 trial assessing pembrolizumab monotherapy, failed to improve OS versus single-drug chemotherapy per investigator’s choice after first-line metastatic TNBC. This underscores the inefficacy of PD-1 inhibitors alone and suggests the association with chemotherapies for the next trials. Indeed, various chemotherapies (such as anthracyclines or taxanes) result in tumor cell death and debris which induce immunogenic cell death. Immunogenic cell death is mediated by damage-associated molecular patterns (DAMPs), promoting tumor phagocytosis and antigen presentation, and may facilitate the induction of a robust antitumor immune response by ICI. In contrast, Keynote-355 showed improvement of PFS and OS in PD-L1-positive metastatic TNBC when chemotherapies including platinum or taxanes were associated with PD-1 inhibitors. This suggests the selection of such chemotherapies combined with ICI for further trials. On an early setting, Keynote-522 showed improved pCR when neoadjuvant and adjuvant pembrolizumab was combined with neoadjuvant carboplatin-paclitaxel followed by anthracycline-based chemotherapy. The IMPassion031 trial combining atezolizumab to nab-paclitaxel followed by atezolizumab with anthracycline-based chemotherapy also significantly increased pCR rate. This further suggests the synergy of ICI with anthracyclines or taxanes. The Impassion130 trial showed increased PFS when atezolizumab was added to nab-paclitaxel chemotherapy in metastatic previously untreated PD-L1-positive TNBC. The trial received accelerated FDA approval which was later withdrawn due to lack of benefit. Accumulating data suggests that immune checkpoint inhibitor antitumor activity is mediated by a non-specific FcγR-mediated modulation of the TME, and not only by the blockade of PD-1/PD-L1 axis on TILs. For example, anti-CTLA4 antibodies may activate FcγR-mediated elimination of intratumoral regulatory T cells (48) and anti-PD-L1 antibodies may activate FcγR-mediated reprogramming of tumor-infiltrating myeloid and NK cells (49). This highlights the role of the Fc domain of therapeutic ICI antibodies in their antitumor activity. The albumin found in nab-paclitaxel (albumin-bound paclitaxel, Abraxane) may prevent the non-specific binding of the ICI antibodies (through Fcγ receptors). Indeed, ‘‘blocking solutions’’, such as bovine serum albumin, are commonly used in laboratories to prevent such non-specific antibody bindings. This might explain the lack of antitumor activity of ICI associated with nab-paclitaxel and suggest replacing nab-paclitaxel by other chemotherapies in the next clinical trials. The involvement of nab-paclitaxel in parallel with atezolizumab in a neoadjuvant setting, such as in the neoTRIP trial, also showed disappointing results up to now, regarding pCR in TNBC patients. In the IMPassion131 trial, adding atezolizumab to paclitaxel did not significantly improve PFS, even in the PD-L1-positive TNBC patients. It should be noted that, according to *post hoc* analyses, most patients were administered corticosteroid premedication throughout paclitaxel therapy, which might alter immunotherapy efficacy. Nevertheless, although the Kaplan-Meier curves remained overlapping for the first 7 months, the trend began to diverge thereafter, favoring the atezolizumab arm and thus, suggesting that the impact of ICI may begin later on, and therefore the need for a longer follow-up.

Regarding HER2-positive BC, HER2-directed monoclonal antibodies, such as trastuzumab, already significantly improved HER2-positive BC prognosis and are probably potent tools for next-generation immunotherapies. Indeed, these antibodies have multiple mechanisms of action, as described above. Therefore, the addition of ICI to trastuzumab/pertuzumab seems to be a promising combination that can at the same time target the tumor cells (through antibody-dependent cellular cytotoxicity and antibody-dependent cellular phagocytosis by anti-HER2 therapy) and trigger myeloid and lymphoid compartment activation (through anti-HER2 therapy and immune checkpoint inhibitor). Associated chemotherapies may further promote tumor immunogenicity. For example, in a metastatic setting, the phase 1b/2 PANACEA trial showed encouraging results, although associated usual chemotherapies were unfortunately missing and should be investigated. ADC are expected to deliver a toxic payload specifically to antigen-expressing-tumor cells. However, ADC have also been clinically shown to be effective in low antigen expression, whereas trastuzumab is not effective. This highlights other mechanisms of action of ADC which might be antigen-independent, as described above. While these mechanisms of action are not yet clear today, combination therapies including ADC are even more difficult to suggest. In phase 2 KATE2 trial, investigating TDM-1 combined with atezolizumab, there is a trend in increased PFS in favor of atezolizumab only in the PD-L1-positive tumors. Adding chemotherapy to ADC and ICI might facilitate the inflammatory response. On the other hand, chemotherapy might suppress immune cells from the TME and by this way alter this inflammatory response. Clinical trials combining ADC with chemotherapy and/or ICI are ongoing (NCT04538742)(NCT04556773) and will first have to assess the adverse events of such combinations. In early HER2-positive BC disease, phase 3 IMpassion050 trial has investigated the association of atezolizumab to anthracycline-based chemotherapy followed by paclitaxel, trastuzumab and pertuzumab in a neoadjuvant setting. Although the primary endpoint has not been reached (pCR was not improved), the neoadjuvant period was short. We should certainly wait a bit longer to observe the known long-term impact of immunotherapy by checking EFS – which is a secondary end point of this study. Indeed, following the administration of the treatments, the mature dendritic cells migrate to the draining lymphoid organs, activate antitumor tumor T cells, which proliferate and go back to the tumor site to kill specifically the cancer cells. The common concept of pCR, which is associated with an important prognosis factor after chemotherapy, might therefore be very different in an immunotherapy setting and we are not sure if pCR is a proper primary endpoint to choose. To optimize the trial design, we would suggest adding atezolizumab to taxanes (with or without platins) and HER2 blockade (monotherapy or dual therapy) neoadjuvant sequence, and not to the anthracycline-based neoadjuvant chemotherapy sequence. Indeed, this may allow the combination of chemotherapy, anti-HER2 blockade and ICI in parallel, whose mechanisms of actions might synergize.

In luminal BC, fewer trials involving immunotherapies have been performed so far. While the first trials were disappointing, many results of ongoing trials are awaited. In high-risk early-stage luminal HER2-negative BC, the pCR rate increased from 13% to 30% in the phase 2 adaptively randomized I-SPY2 trial by adding pembrolizumab to neoadjuvant paclitaxel chemotherapy followed by anthracycline-based chemotherapy. This was associated with a high EFS rate of 93% at 3 years. This chemo-immunotherapy combination is similar to those that are effective in the other BC subtypes, and might succeed in the randomized phase 3 Keynote 756. In metastatic luminal BC, results are encouraging as well (50). While a previous trial adding pembrolizumab to eribulin did not improve PFS or OS in luminal metastatic BC patients pretreated with 2 or more lines of hormonal therapy (51), there are many other combination therapies to investigate. For example, combinations of ICI with aromatase inhibitors and CDK 4/6 inhibitors might synergize. Indeed, CDK 4/6 inhibitors induce the presentation of tumor antigens on dendritic cells, stimulate cytotoxic T cells and suppress regulatory T cells activity (52). The impact of hormone therapies on the antitumor immune response is less clear, but aromatase inhibitors might improve CD8 T cell/regulatory T cell ratio (53).

## New immune checkpoint targets

**Figure 3:**
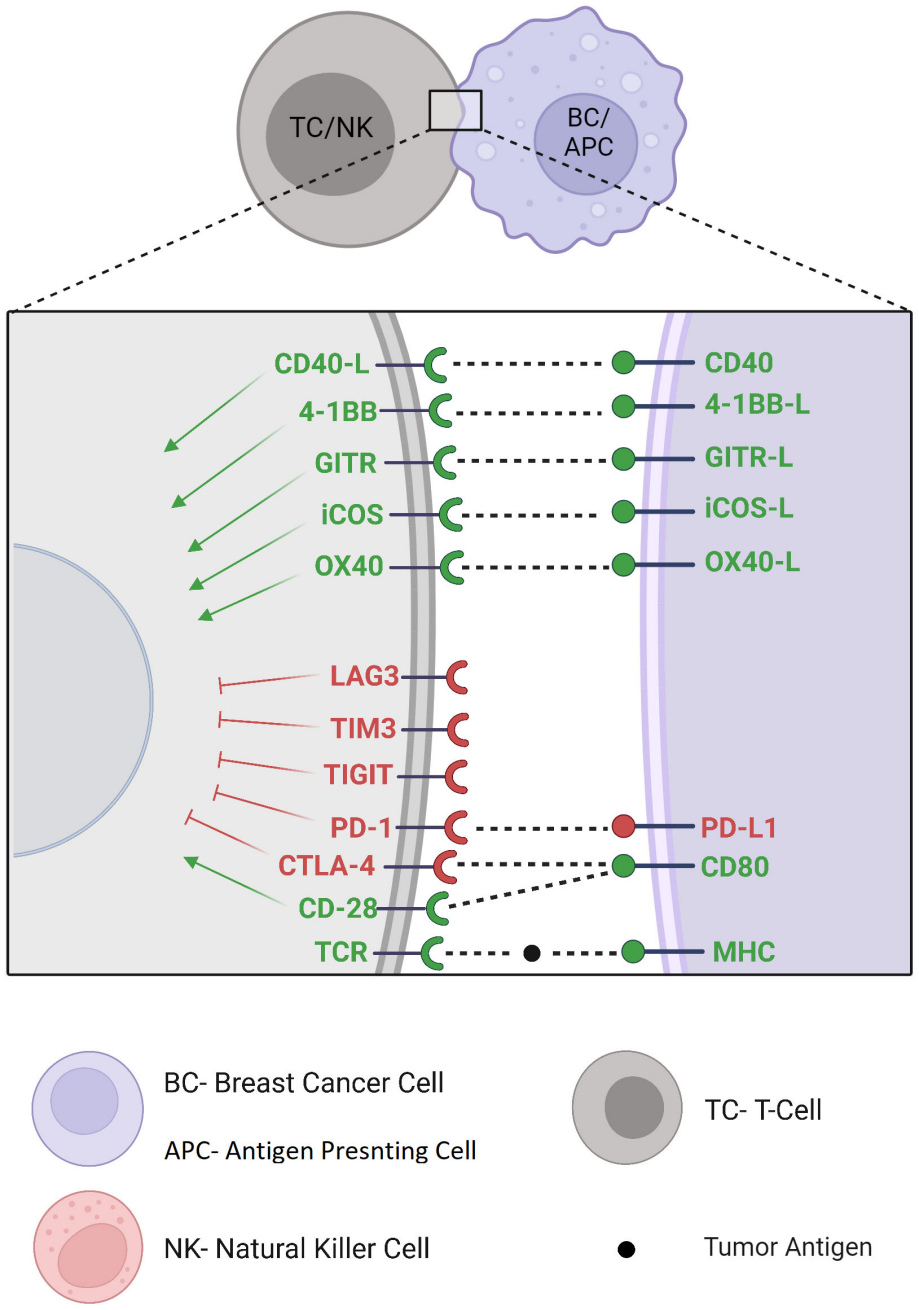
New immune checkpoint targets.

Immune checkpoint discovery has increased interest in other recently discovered inhibitory and stimulatory immune checkpoint pathways and gave rise to new clinical trials with many other potential immune checkpoint targets (**Figure 3**). For example, Lymphocyte Activation Gene-3 (LAG-3) signaling pathway is mainly expressed on lymphocytes where it inhibits T-cell activation, proliferation and cytokine production (54). The combination of eftilagimod α (a monoclonal antibody inhibiting LAG-3) with paclitaxel showed clinical benefit in 90% of metastatic BC patients at 6 months, supporting the future development of this agent in combined first-line regimens (55). Ongoing clinical studies investigate its safety and efficacy alone, associated with other immunotherapies, or through a bispecific antibody targeting ICI and LAG-3 simultaneously (NCT02614833)(NCT03742349)(NCT03849469). T-cell Immunoglobulin and Mucin domain-containing protein 3 (TIM-3) contains multiple co-inhibitory receptors expressed on different types of immune cells (56). TIM-3 antagonistic monoclonal antibodies are currently being clinically investigated in advanced tumors including BC, alone or with a co-blockade with other immunotherapies or chemotherapies (NCT02817633)(NCT04370704)(NCT05287113)(NCT03446040). T-cell Immunoreceptor with Ig and ITIM domains (TIGIT) is restricted to lymphocytes where it exerts its immunosuppressive effect by competing with costimulatory signals such as CD226, like the CD28/CTLA-4 pathway (57). Monoclonal antibodies targeting TIGIT undergo early-stage clinical trials as monotherapy or in combination with current ICI in patients with locally advanced or metastatic malignancies including BC (NCT03628677). V-domain Immunoglobulin Suppressor of T-cell Activation (VISTA) is expressed on myeloid cells, regulatory T cells, and on a lesser extent, on BC tumor cells (58). Preclinical trials of VISTA monoclonal antibody-mediated blockade showed remarkable protective antitumor effects (59). CA-170 is an orally available dual small molecule inhibitor of VISTA and PD-L1 being examined in patients with advanced tumors such as BC (NCT02812875). B7 Homolog 3 protein (B7-H3) displays complex immunomodulatory activity in innate and adaptive immunity with costimulatory as well as inhibitory functions and still requires further elucidation. Its receptor has not yet been identified (60). The blockade of B7-H3 is being investigated in early-stage clinical trials in various cancers including BC (NCT03729596)(NCT04145622)(NCT03406949). OX40 and OX40 ligands are co-stimulatory molecules expressed on different types of immune cells whose interaction leads to T-cell proliferation and decreased regulatory T cells. Although this axis seems to play a controversial role in the antitumor response, many oncological clinical trials are focusing on agonistic antibodies with potential antitumor activity (61) (NCT03971409)(NCT02410512). Inducible CO-Stimulator of T cells (ICOS) is a costimulatory molecule induced by T-cell activation, leading to secondary stimulatory signals. Besides its expression on antitumor effector TILs, ICOS is also expressed on regulatory T cells from the TME, on which it confers an immunosuppressive activity. Since ICOS does not induce a cytotoxic immune response independently (62), clinical investigation of monoclonal antibodies for potential synergistic effects in association with other immunotherapies in advanced malignancies, including BC, are currently conducted (NCT02904226, NCT03447314, NCT03829501). Glucocorticoid-Induced TNFR-Related gene (GITR) activation promotes effector T-cell activity and inhibits tumor-infiltrating regulatory T-cell function. GITR agonism alone does not seem to be sufficient to induce a significant clinical response to therapy. However, there is a rationale for reinvigoration TILs exhaustion through combination with PD-1 blockade (63), which is currently under clinical investigation in advanced malignancies including BC (NCT02628574)(NCT03126110). 4-1BB is a powerful costimulatory signal whose agonistic stimulation on CD8+ T-cells enhances survival, function and memory differentiation in preclinical models (64). Anti-4-1BB agonist monoclonal antibodies are under clinical investigation in combination with ICI in patients with locally advanced or metastatic TNBC (NCT02554812)(NCT03971409) and HER2-positive BC (NCT03414658). A bispecific antibody targeting HER2 and 4-1BB has also shown encouraging data of safety and clinical benefit in phase 1 clinical trial (65) (NCT03330561)(NCT03650348). In addition, 4-1BB upregulation through Fcγ receptor stimulation on NK cells (66) may suggest a synergy between 4-1BB agonism and anti-HER2 blockade/anti-HER2 ADC, which is also currently investigated (NCT03364348).

CD40 is mainly expressed on antigen-presenting cells, where ligation by CD40 ligand results in antigen-presenting cell activation and therefore priming and activation of effector T cells (67). Agonist monoclonal antibodies are under investigation, as monotherapy or in combination, in patients with advanced malignancies including BC (NCT03329950)(NCT05029999).

## Adoptive cellular therapy

**Figure 4:**
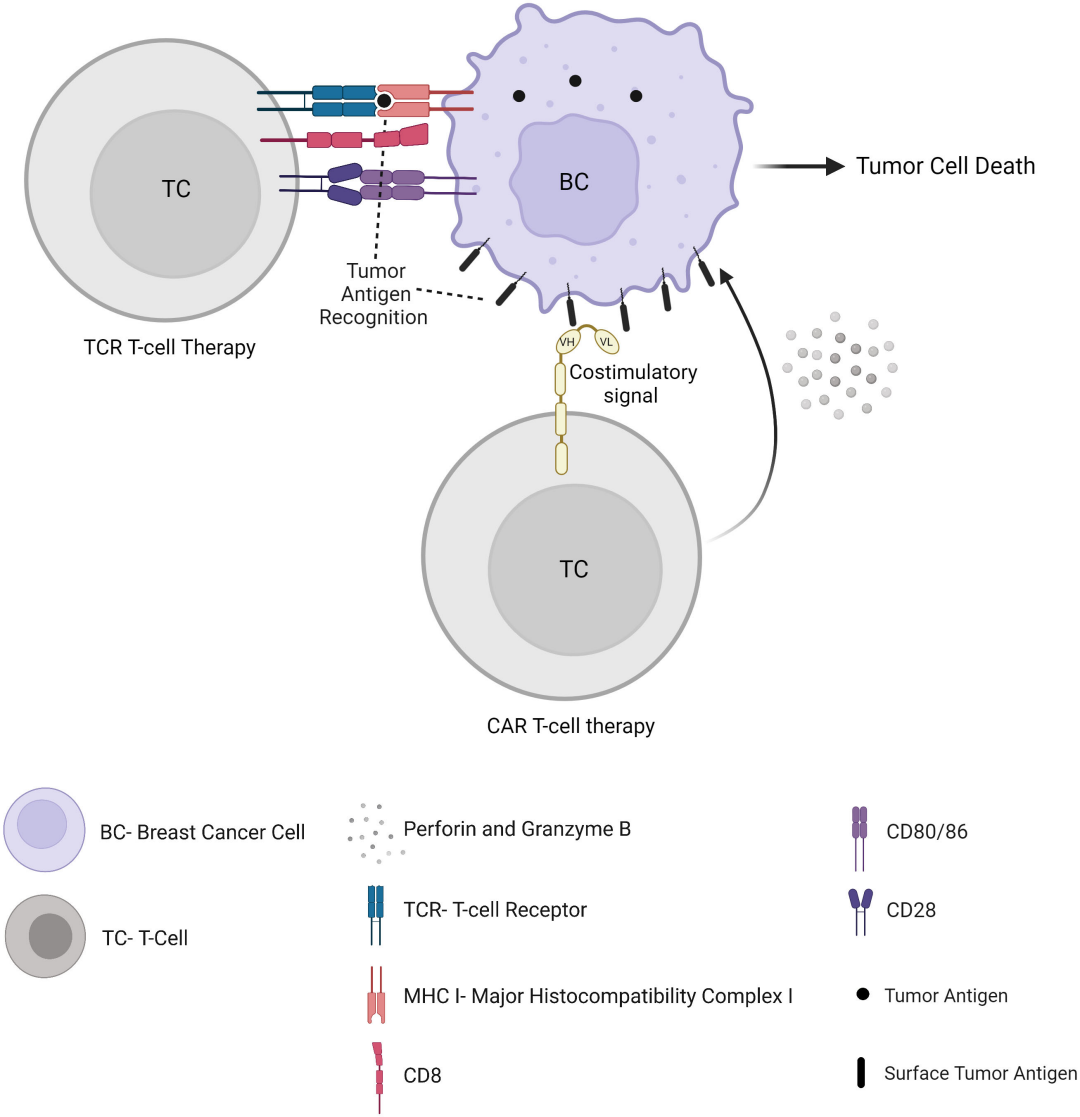
Adoptive cellular therapy.

Despite their antitumor reactivity, TILs, if they are not suppressed within the TME, are often exhausted, contributing to tumor immune escape mechanisms. Autologous TILs can be isolated from tumor specimens, expanded, and activated *in vitro*, and reinfused into the patient, alone or in combination with interleukins. In TCR-engineered lymphocytes and CAR T cell therapy, the cells are isolated from a patient’s peripheral blood through leukapheresis, and genetically modified to express either a T cell receptor (TCR) or a chimeric antigen receptor (CAR) (**Figure 4**). Unlike its success in haematological malignancies with FDA’s approval of tisagenlecleucel and axicabtagene ciloleucel, adoptive cell therapy in solid tumors is associated with major obstacles. This includes the identification of appropriate specific tumor antigen targets and the presence of a strong immunosuppressive TME. However, numerous adoptive cellular strategies have recently been developed to fight solid tumors and are under investigation in preclinical studies and clinical trials, including in BC patients. Once expressed in T cells and accompanied by costimulatory domains (such as 4-1BB or CD28), CAR T cells display antigen-specific recognition, activation, proliferation and cytotoxic function, independent of MHC presentation, which is a major advantage compared to TCR therapy (see hereunder) (68). Several targets have been proposed to date, such as mesothelin (69) (NCT02792114)(NCT02414269), c-Met (70), CEA (NCT04348643), EPCAM (NCT02915445), CD70 (NCT02830724), or MUC1 (NCT04020575)(NCT04025216). Clinical trials have also been started with an infusion of CAR T-cells targeting HER2 (NCT04511871)(NCT04430595)(NCT03740256), including intraventricular administration in patients with brain metastases (NCT03696030). CAR-T cells could also be designed to recognize a universal motif such as an Fc portion of immunoglobulins (71), allowing a potential antitumor activity in synergy with antibody treatments such as trastuzumab. Besides, other molecules demonstrated *in vitro* and *in vivo* antitumor activity against BC, such as Natural killer group 2, member D (NKG2D) (72) or Receptor tyrosine kinase-like orphan receptor 1 (ROR1) (73). CAR-T cells targeting these molecules will probably be clinically investigated soon as well.

Although CAR T-cell therapy has attracted major interest, TCR therapy can target both surface and intracellular proteins whose peptides are presented onto MHC molecules (**Figure 4**). As a result, TCRs could target more tumor antigens and be more tumor-specific (74). In a metastatic HER2-overexpressing BC patient, an adoptive transfer of autologous HER2-specific cytotoxic T cells has been shown to lead to accumulated T cells in the bone marrow along with a loss of bone marrow-residing disseminated tumor cells (75). The adoptive transfer of allogeneic T cells in metastatic BC patients suggested their contribution to a transient early tumor response (76). TCR T-cell therapy is currently being evaluated in patients with metastatic or locally recurrent and unresectable disease including BC through tumor-associated antigen-specific cytotoxic T lymphocyte infusion (NCT03412877)(NCT03093350).

In addition, several clinical studies investigating TIL therapy in BC have been performed. A phase 2 clinical trial demonstrated a case of adoptive transfer of autologous mutant-protein-specific TILs in association with pembrolizumab and interleukin-2 that led to the complete and durable regression of metastatic disease in an HR-positive BC patient who failed to respond to multiple previous lines of therapy (77). Clinical trials are currently investigating the transfer of autologous TILs in patients with pretreated metastatic BC (NCT01174121)(NCT04111510).

Lastly, a growing interest in CAR-NK cell immunotherapy (78) has demonstrated promising preclinical studies in TNBC. In the future, personalized immunopeptidomic profiling of the tumors may allow us to identify new potential therapeutic target antigens (79). Next-generation engineering CAR-T cells may also target several aspects of the TME to treat solid tumors such as the tumor stroma and vasculature as opposed to tumor cells alone, as it has recently been suggested *in vivo* (80). In addition, next-generation CAR-T cells endowed with bispecific CAR dual specificity targeting multiple tumor antigens have been shown to potentiate the antitumor activity in tumor models and to minimize parallel reactivity against normal tissues harboring single antigen, which could present a novel approach focusing CAR-T cells to tumor cells (81).

## Bispecific antibodies

**Figure 5:**
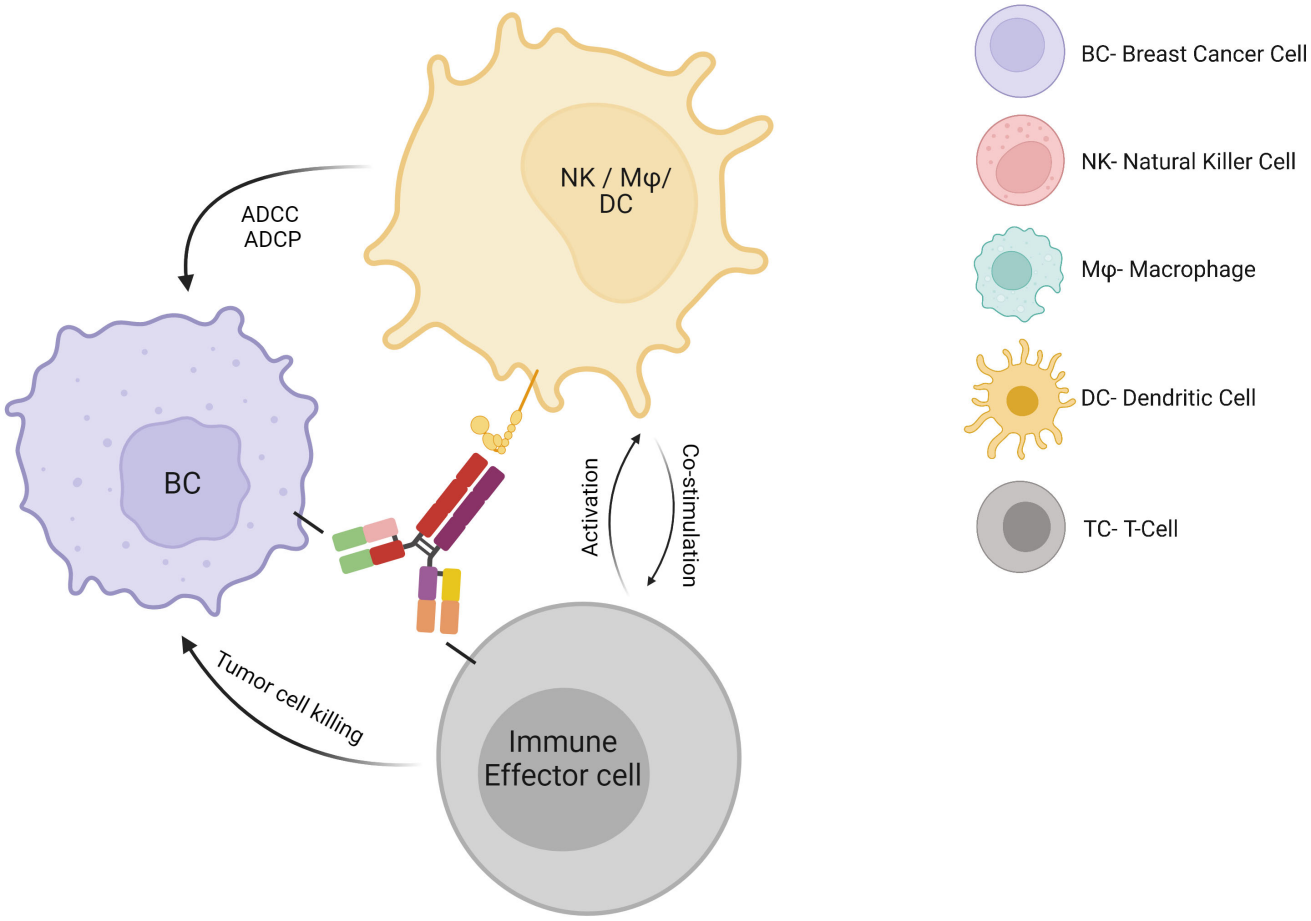
Specific elimination of tumor cells by bispecific antibody.

With FDA’s approval of blinatumomab in the treatment of B-cell malignancies, recent efforts have focused on the extension of multifunctional bispecific antibodies to BC immunotherapy. Most bispecific antibodies for cancer immunotherapy consist of two arms. One arm binds tumor-associated antigens on cancer cells. The other arm binds effector immune cells, with selective binding to activate Fcγ receptors, resulting in the formation of a tri-cell complex and specific elimination of tumor cells (**Figure 5**). This elimination is independent of TCR specificity, co-stimulatory signals, or peptide antigen presentation, representing a major advantage of bispecific antibodies in cancer immunotherapy. Compared to trastuzumab, it has been shown that these trifunctional antibodies mediate the elimination of tumor cells expressing HER2/neu at low levels, which was associated with a Th1-based cytokine release (82) and a potent antitumor activity (83). Ertumaxomab which targets CD3 and HER2 simultaneously has shown encouraging results in phase 1 clinical trials, with an antitumor response seen in 30-59% of metastatic BC patients, especially in HER2-positive BC patients (84). This provides a strong rationale for further studies involving unlimited combinations of bi- or tri-specific antibodies in the therapeutic strategies against BC. Consecutively, new phase 1 and 2 clinical trials evaluate the efficacy of bi- or tri-specific antibodies in locally advanced or metastatic BC patients, through for example HER2/CD3 bispecific target (NCT03448042), NK cell/T-cell/HER2 tri-specific target (NCT04143711), HER2/PD-1 bi-specific target (NCT04162327), or two non-overlapping domains of HER2 target (NCT04224272). A multitude of other bispecific antibodies are designed to simultaneously target molecules on the tumor cells and/or the TME, such as different immunosuppressive pathways (85) or cell-cell adhesion molecules overexpressed on BC tumor cells (86).

## Therapeutic peptide and protein-based cancer vaccines

**Figure 6:**
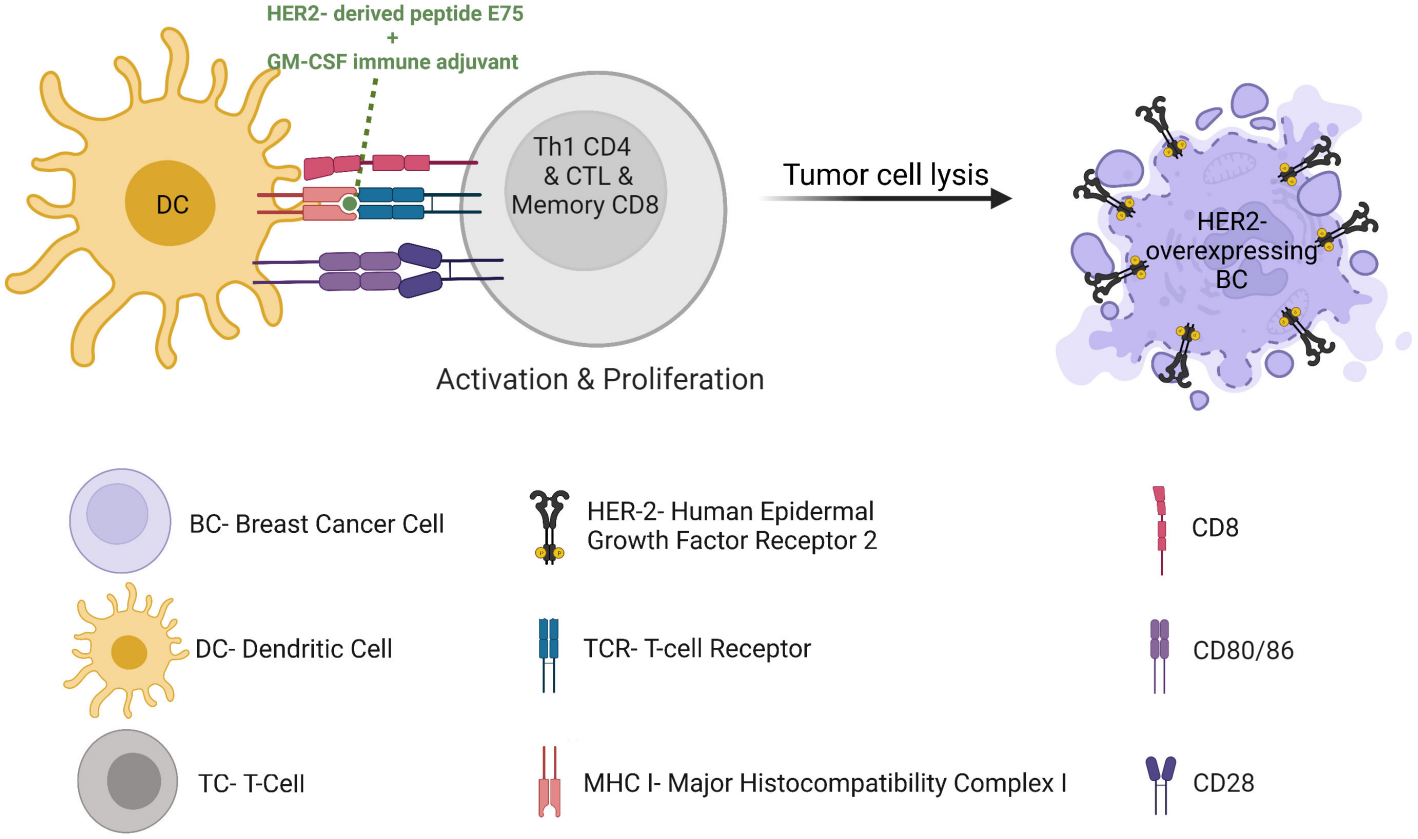
HER2-positive breast cancer vaccine.

In the age of cancer immunotherapy we see a renewed interest in harnessing cancer-targeting vaccines for therapeutic purposes. Such vaccines can be preventive or therapeutic. Several preventive vaccinations are FDA-approved, such as HPV-related genital/head and neck cancers, and HBV-related hepatocellular carcinoma. Among the FDA-approved therapeutic cancer vaccines today are the Bacillus Calmette-Guérin (BCG) for early-stage bladder cancer and Sipuleucel-T (Provenge) for the treatment of metastatic castrate-resistant prostate cancer. No vaccine has been approved for clinical use in BC to date. However, a rising interest for the development of peptide vaccines has been seen in recent years. For example, the most studied cancer vaccine which has been successful to date is the E75 HER2 peptide vaccine, also known as Nelipepimut-S when combined with Granulocyte-Macrophage Colony-Stimulating Factor (GM-CSF). E75 is a small peptide derived from the HER2 receptor which is expected to bind HLA molecules and therefore activate cytotoxic effector T cells (**Figure 6**). This vaccine demonstrated in high-risk HLA-A2 BC patients a recurrence rates of 5.6% in vaccinated patients compared to 14.2% in control unvaccinated participants at 20 months follow-up (87). However, the observed difference could not be repeated in later analyses, possibly due to a lack of immune re-boost. Alternatively, one could speculate that the vaccine could be more efficient with trastuzumab or with an associated myeloid-compartment targeting strategy. Consequently, a phase 1/2 clinical trial assessed the administration of nelipepimut-S and GM-CSF in high risk BC patients in the adjuvant setting, with booster inoculations every 6 months until trial completion at 5 years. This trial demonstrated prevention of disease recurrence at 94.6% in optimally vaccinated patients versus 80.2% in the control group, with minimal toxicities (88). Unfortunately, the phase 3 study was discontinued due to futility (89). However, the addition of trastuzumab to nelipepimut-S and GM-CSF has shown a DFS of 92.6% compared with 71.9% for trastuzumab and GM-CSF group in a phase 2b clinical trial in patients with TNBC (90), with no additional overall or cardiac toxicity compared with trastuzumab alone. Together, this suggests a synergy between trastuzumab and the HER2 peptide vaccine and highlights the importance of selecting the most relevant arms when designing a clinical trial. In addition, it highlights the fact that most BC patients are defined as HER2-negative tumors following fluorescence *in situ* hybridization (FISH) amplification methods, though they display some HER2 expression by immunohistochemistry (IHC) and therefore might respond to anti-HER2 therapies in some combination therapies. In HER2-positive BC patients, a possible synergistic immunologic effect of nelipepimut-S and trastuzumab is currently being investigated (NCT02297698). A meta-analysis of randomized clinical trials suggested significant benefits to vaccination over control, though the high heterogeneity of the patients treated in the involved trials led to unclear final results (91). Moreover, the trials generally show low toxicity of therapeutic vaccination. Vaccination thus remains an attractive strategy that seems to promote both effector immune response and protective memory immunity potentially controlling tumor relapse. Preventive BC vaccines are therefore also under investigation in patients in remission to prevent or delay relapse (NCT02780401)(NCT03384914). Additional HER2-derived peptide cancer vaccines have demonstrated beneficial impacts on BC antitumor immunity and clinical outcome, such as GP2 and AE37 (92), folate receptor α peptide vaccine (93) (NCT03012100), sialyl-Tn (sTn) conjugated to keyhole limpet hemocyanin (KLH) (94), and oxidized mannan-MUC-1 vaccine (95). STn-KLH vaccine (an epitope found among others on MUC1 that activates estrogen receptor-α function), given concurrently with endocrine therapy, offered a robust antibody response to the vaccine and an OS advantage to metastatic BC patients in a retrospective blinded review involving 1028 women (96). This further justifies prospective randomized trials combining anti-MUC1 vaccine with endocrine therapies. Unlike peptide-based cancer vaccines, protein-based cancer vaccines have not been explored to the same extent up till now. However, clinical studies have suggested safety, immunogenicity, long-term survival and a few high grade adverse events of protein-based cancer vaccines in patients with HER2-overexpressing BC refractory to trastuzumab (97). They are under clinical investigation in BC patients undergoing neoadjuvant endocrine therapy (NCT02204098) and in combination with ICI (NCT03632941). Thanks to the next-generation sequencing of tumor mutations and epitope-prediction strategies, new patient-specific neoantigen-based cancer vaccine will probably allow inducing highly specific antitumor immune responses (NCT02316457).

## Autologous tumor cells

**Figure 7:**
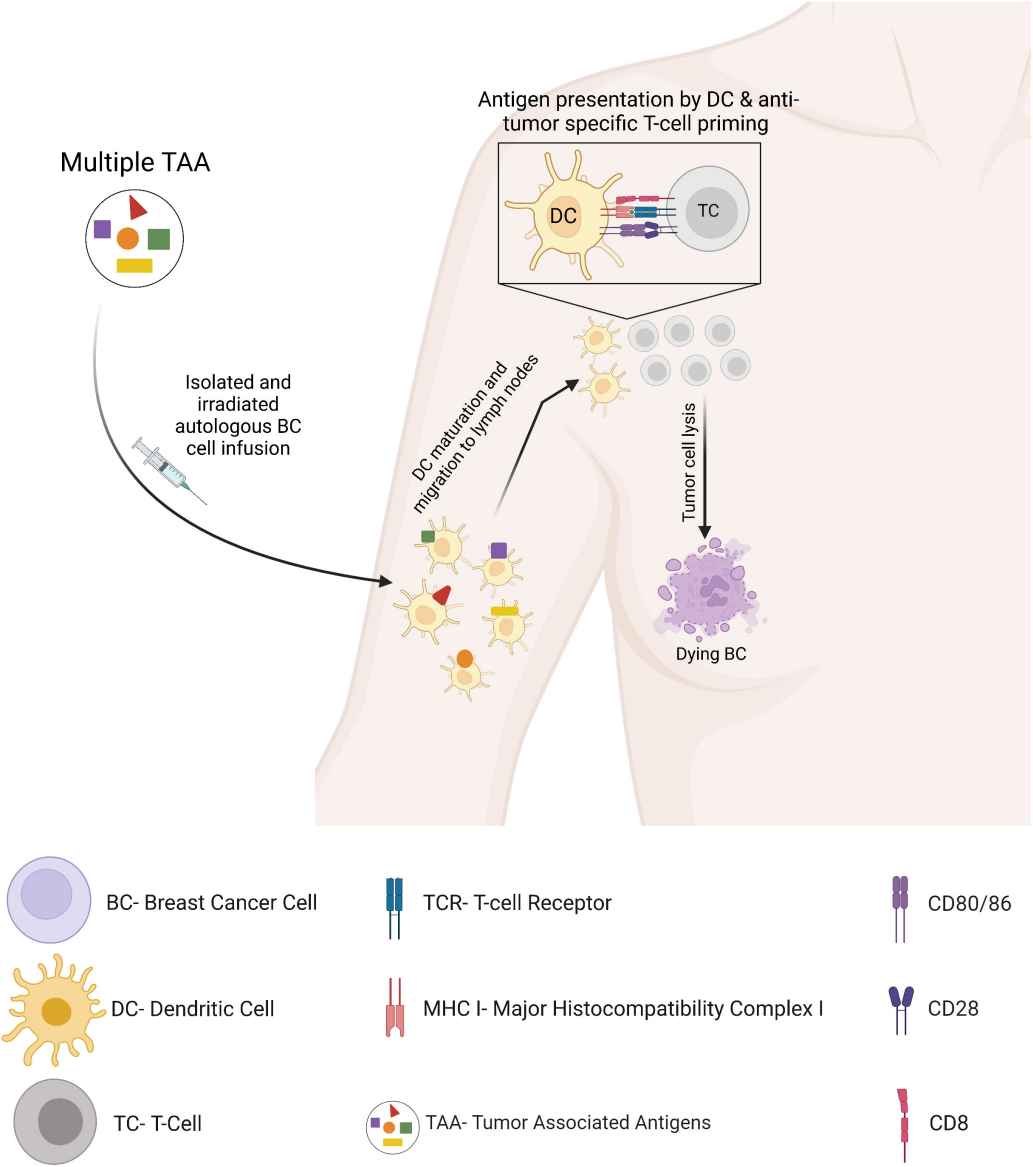
Autologous tumor cell vaccine.

Personalized vaccines targeting patient-specific mutated neo-antigens, through autologous tumor cells, alone or pulsed on dendritic cells, are new potential strategies (98). Autologous tumor cell-based cancer vaccines, obtained from tumor cell isolation, may overcome the difficulties of selection of an appropriate tumor-associated antigen. These tumor cells harbor a complete and individualized repertoire of tumor-associated antigens, therefore potentially triggering a polyclonal T-cell response against various tumor cells (99) (**Figure 7**). Several translational and clinical trials undergone in BC patients have suggested that autologous tumor cell-based vaccines may be effective and safe, even among patients with depressed immunity (100). BC cell lines can also be used and have shown the induction of a humoral and T cell-mediated immune response alone or in combination with low-dose chemotherapy or costimulatory molecules, although the clinical results showed limited success. This is possibly because the cell lines do not express the antigen repertoire of the tumor because of inter- and intra-tumoral heterogeneity among cancer patients (101). This obstacle may be overcome by an autologous tumor cell-based vaccine, which may also minimize tumor immune escape through antigen loss observed in clinical trials (102). In this line, autologous BC tumor cells harvested from stage II-III and metastatic BC patients, irradiated and reinfused, led to encouraging results (103). Vaccination using irradiated, genetically modified GM-CSF-secreting tumor cells has shown to induce an enhanced antitumor immunity in a phase 1/2 clinical trial alone or in association with chemotherapy in metastatic BC patients (104). In an additional clinical trial, an irradiated BC cell line, endowed with antigen-presenting cell activity and in association with interferon-α, showed in heavily pretreated advanced BC patients an objective tumor regression in parallel with a decrease in circulating cancer-associated cells, with no serious adverse events (105). Unfortunately, clinical data about autologous tumor vaccination in BC is still limited to date, despite its promising antitumor immune potential.

## B cell-based vaccines

Trastuzumab’s success led to enthusiasm for B cell-based vaccines, which induce an endogenous active and specific humoral B cell-immune response (antibodies with antitumor activity) from the patient’s own B cells. The antibodies secreted in the patients are similar to those of the drug itself, and may offer a promising alternative to antibody administration. Indeed, trastuzumab antibody yield seems to be a major challenge for large-scale production. Trastuzumab is usually produced in Chinese Hamster Ovary Cells in incubators of 80 up to 12 000 liters, which makes a year of treatment per person very expensive. HER-2 B-cell peptide vaccine already demonstrated a robust production of anti-HER2 antibodies in patients with advanced or metastatic HER2-positive cancer of the stomach or the gastroesophageal junction. Besides, there is a correlation between the levels of anti-HER2 antibodies and the clinical response to therapy. In addition, the early data indicated a benefit in OS (hazard ratio of 0.418) with no added toxicity, in association with standard-of-care chemotherapy, compared to chemotherapy alone (106). The combination of two peptide B-cell epitope vaccines, representing trastuzumab and pertuzumab binding sites, showed safety and antitumor activity performed in patients with advanced solid tumors including BC (107). Other clinical trials are ongoing (NCT01376505). The next generation of clinical studies will have to involve the combination of vaccines with ICI, since preclinical data suggest synergistic mechanisms of action. Indeed, IFNγ secreted by cytotoxic antitumor T cells upregulates PD-1/PD-L1 axis-mediated immune suppression. The addition of ICI to vaccine administration may block the induced immunosuppressive feedback and allow the induction of robust antitumor immunity (108).

## Dendritic cell-based vaccines and TLR agonists

**Figure 8:**
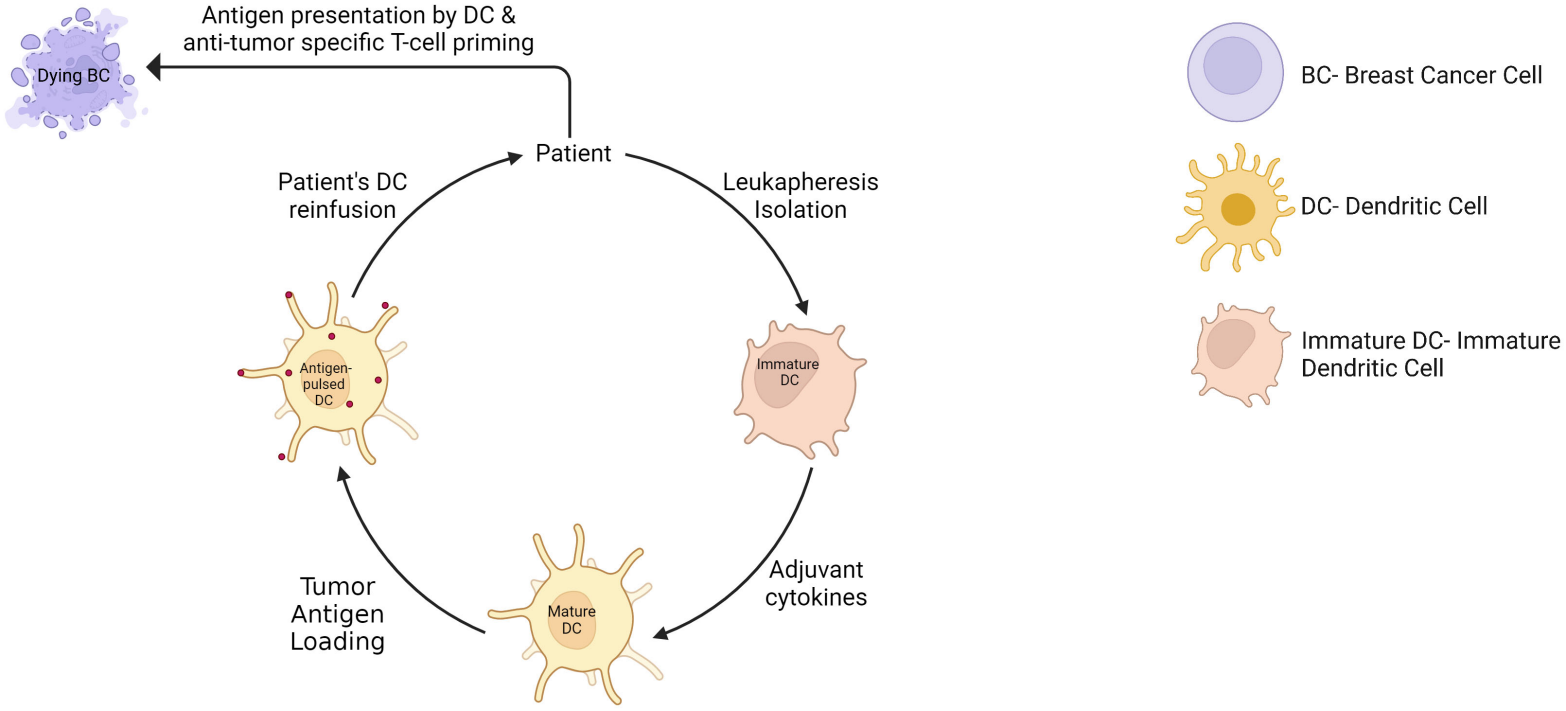
Dendritic cell vaccine therapy.

Whole-cell dendritic cell (DC) vaccines are tumor-specific DC infusion, mainly isolated from patients’ peripheral blood monocytic cells, exogenously matured and expanded using various cytokines, and loaded with tumor lysate or antigens (**Figure 8**). This approach has shown therapeutic potential with limited toxicities and is extensively investigated in clinical trials. For example, DC loaded with tumor antigens, in association with chemotherapy, have been shown to significantly increase PFS and OS in metastatic BC patients compared to chemotherapy alone over a 10-year follow-up (109). Another clinical study showed a prolonged PFS from 31% to 76.9% with DC vaccine in ER/PR double-negative stage II/III BC patients (110). In addition, the DC vaccine added to neoadjuvant chemotherapy increased the pCR rate to 28.9% compared to 9.09% in the control group with neoadjuvant chemotherapy alone (111). Remarkably, these results were observed in the PD-L1-negative population, and may probably be explained by a poorly immunosuppressed TME where the vaccine can trigger an appropriate antitumor activity. This supports again the hypothesis that the combination of ICI to cancer vaccine might provide a synergistic antitumor activity. Lapuleucel-T, consisting of an adoptive transfer of autologous antigen-presenting cells activated *in vitro* with a recombinant fusion protein comprising HER-2 sequences, demonstrated safety and significant antitumor immune response in patients with HER-2-overexpressing metastatic BC, associated with clinical response or disease stabilization in some patients (112). Another example is the vaccination with p53 peptide-pulsed DCs, which showed disease stabilization of advanced BC patients, and a correlation between p53 expression of tumor cells and the induction of a p53-specific T cell response (113). Other clinical trials are currently investigating DC vaccine in BC patients, such as HER2-pulsed DC vaccine (NCT02063724) an multiepitope DC vaccine (NCT00266110), and are also investigated in a neoadjuvant setting (NCT02018458)(NCT02061423)(NCT03387553).

Human DC cells express Toll-Like receptors (TLRs) which play a key role in recognizing signals from the microenvironment and adapting accordingly. The DCs can be targeted and activated through TLR agonists which have shown promise in preclinical and clinical cancer studies, enhancing antitumor T-cell response. Several clinical trials are currently evaluating TLR agonists. For example, topical TLR-7 agonist imiquimod, in association with nab-paclitaxel, has shown an immune-mediated disease regression in treatment-refractory BC chest wall metastases in phase 2 clinical trial (114). Systemic TLR7 agonists are also under development to activate the antitumor immune response in patients with advanced cancer (115). Therapeutic cancer vaccination using TLR3 agonists, such as PolyICLC, are also currently under investigation in patients with advanced cancer including BC (NCT02643303). Systemic DC expansion is also being tested in patients with cancers including metastatic BC, through vaccination with polyICLC in association with Flt3L (NCT03789097).

## DNA/mRNA-based cancer vaccines

The favorable clinical experience observed with COVID-19 vaccine may help facilitate research and development in the field of DNA/mRNA-based cancer vaccines. These vaccines are mainly viral replicon particles, or completely synthetic lipid nanoparticles, and contain the genetic information coding for tumor-specific antigens or tumor-associated antigens. Upon administration, the DNA is transcribed into mRNA, which is then translated to synthesize peptides and proteins within the ribosomes. This leads to the presentation of the peptides onto HLA, ultimately activating highly specific cytotoxic and memory T cells against tumor, possibly for a longer period of time compared to peptide or protein-based cancer vaccines. A phase 1 clinical trial investigating a naked plasmid DNA vaccine in metastatic BC patients is currently ongoing (NCT02204098) and preliminary evidence suggested an increased specific CD8+ T-cell proliferation and cytotoxicity along with an improved PFS rate (116). Another phase 1 clinical trial of 66 BC patients with advanced HER2-positive disease investigated the administration of a plasmid DNA coding for HER2 molecule, associated with GM-CSF as an adjuvant, for 3 immunizations. This study demonstrated the induction of an anti-HER2 immunity in most patients, persisting after the end of the vaccinations, and safety with 10-year postvaccine toxicity assessments (117). Importantly, the authors underscore the fact that high anti-HER2 immunity is associated with favorable clinical outcomes after trastuzumab therapy. In line with these results, a randomized phase 2 trial is currently in progress (117). Moreover, in TNBC patients with residual disease after neoadjuvant chemotherapy, another phase 1 clinical trial has indicated that DNA vaccine induced neoantigen-specific immune response in 88.8% of the patients along with a PFD of 87.5% in the vaccinated patients, compared to 49% in historical controls (118). Adverse events were mainly injection site reactions and other limited toxicities. The administration of a mRNA-based vaccine encoding a portion of HER2 (VRP HER2), with or without an additional anti-HER2 targeted therapy, also demonstrated stable diseases and partial response in a phase 1 clinical trial with advanced HER2-positive BC patients (119). In this study, PFS correlated with perforin expression by memory T cells. Other clinical trials investigating DNA/mRNA-based vaccine immunotherapies are ongoing, for example in advanced and metastatic BC patients (NCT02157051), with concurrent ICI (NCT03632941), or in non-metastatic, node-positive BC patients who are in remission (NCT02780401).

## ℽδ T cells

In contrast to conventional αβ T cells, ℽδ T cells are a distinct little subset of T cells, which typically recognize and kill malignant cells rapidly and independently of HLA restriction. This highlights the antitumor impact that ℽδ T cells may play in the next generations of cancer immunotherapies, for example through adoptive transfers of allogeneic cells from healthy donors rather than autologous ones. In addition, ℽδ T cells contribute also indirectly to the antitumor immune response by communicating with other tumor-infiltrating immune cells (120). Vℽ9Vδ2 T-cell therapy has demonstrated safety and higher survival of late-stage lung or liver cancer patients (121). In BC, although ℽδ T cells have been suggested to display a degree of functional plasticity with possible opposing effects on the growth of breast tumors (122), the antitumor potential of these cells makes them an encouraging immunotherapeutic tool to exploit as well. Indeed, ℽδ T cells were correlated with an improved pCR rate following neoadjuvant therapy, and an improved DFS and OS (11). In addition, the antitumor activity demonstrated by the use of bisphosphonates in some BC clinical trials may be at least partly due to ℽδ T cell activation (123). Furthermore, BC-infiltrating ℽδ T cells have been suggested to be involved in the efficacy of trastuzumab, through a mechanism of ADCC (124). ℽδ T cells have also been associated with remission in TNBC patients (125), although conflicting observations have been reported regarding a potential suppressive function of ℽδ T cells on αβ T cells (126).

For example, a phase 1 clinical trial was conducted in which the bisphosphonate zoledronate, a Vℽ9Vδ2 T-cell agonist, plus low-dose interleukin-2 (IL-2), were administered to terminal advanced metastatic BC patients. The treatment was well tolerated by all patients. In addition, the patients who showed a robust peripheral population of Vℽ9Vδ2 T cells had declining CA15.3 levels and displayed partial remission and stable disease, compared to patients who failed to sustain Vℽ9Vδ2 T cells (127). Infusions and other agonists of Vℽ9Vδ2 T cells are investigated in clinical trials (128) (NCT03183206)(NCT02781805) as monotherapy or in combination with an ICI (NCT04243499). Furthermore, we expect that adoptive transfers of engineered T cells expressing antitumor ℽδ TCR will also be investigated in BC patients in the upcoming years (129). Although most clinical results have not shown encouraging results thus far, these attractive cells must be better understood and will then certainly show us their hidden secrets, hopefully to the advantage of BC immunotherapy.

## Tumor-associated macrophages

**Figure 9:**
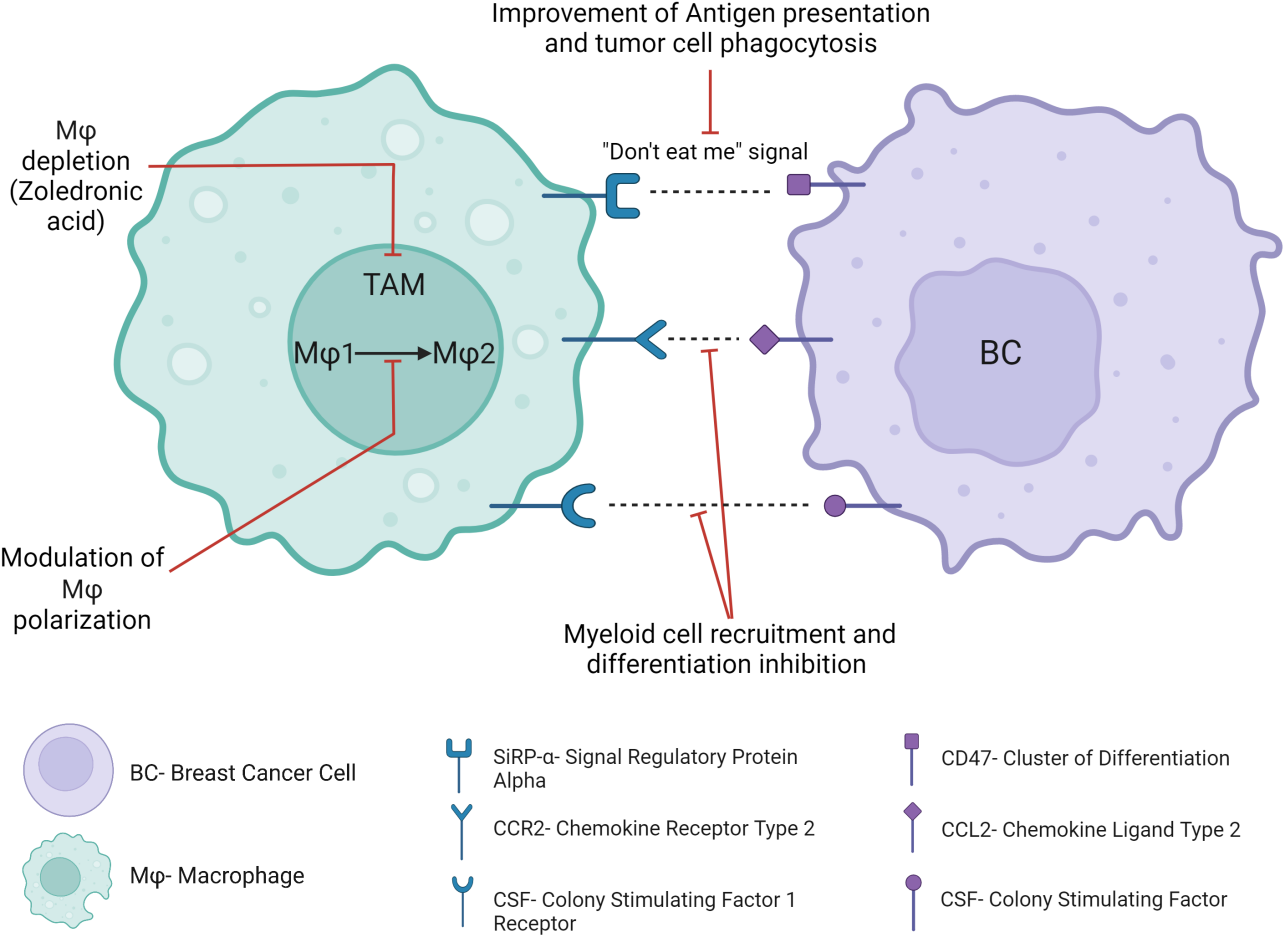
Antitumor strategies targeting tumor-associated macrophages.

Macrophages are among the most abundant immune cells within the TME, where they play a key role in the antitumor immune response through pro-inflammatory macrophage activation, tumor cell phagocytosis and antigen presentation. Clinically approved cancer therapies such as trastuzumab exert at least partially their effects through these mechanisms (130), while pre-clinical studies attempt to optimize these mechanisms (131). Experimental mouse models and clinical studies demonstrated that macrophages are educated by the TME and generally adopt protumoral and immunosuppressive functions, in contrast to their tumoricidal role following *in vitro* activation (132). A meta-analysis of BC patients demonstrated that Tumor-Associated Macrophages (TAMs) were significantly correlated with an aggressive phenotype, metastasis and poor clinical prognosis (133), lack of pCR, resistance to chemotherapy (134) and tamoxifen (135). Furthermore, the protumoral effect of TAMs is often further reinforced after cancer treatments, through macrophage recruitment and polarization, limiting the efficacy of chemotherapy and radiotherapy and promoting early tumor recurrence (136). Clinical trials investigating TAMs-targeting strategies (**Figure 9**) suggest potential impact in the treatment of cancer.

Blocking SIRPα on the surface of TAMs and DCs, which blocks its interaction with CD47 on the surface of tumor cells and their subsequent “don’t eat me” signal, restores the phagocytosis of tumor cells. Clinical trials suggested safety and therapeutic potential (137) in several cancer patients. Furthermore, the concomitant blockade of the CD47/SIRPα axis with tumor-targeting monoclonal antibodies such as trastuzumab may provide a synergistic phagocytic antitumor activity. In this way, the preliminary antitumor activity of this association has been demonstrated in rituximab-refractory non-Hodgkin lymphoma patients and in solid tumors (138, 139). In BC, no clinical trial has assessed such a combination up to now. However, CD47 gene expression has been found to limit the therapeutic activity of trastuzumab in HER2-positive BC patients (15). Other “don’t eat me” anti-phagocytic signals include PD-L1, or recently highlighted CD24, which has been recently suggested as a new therapeutic target for BC immunotherapy (140). Other potential myeloid immunotherapeutic strategies may be the blockade of myeloid cell recruitment to the TME, differentiation into TAMs or proliferation within the TME. For example, CCR2-CCL2 axis contributes to metastatic BC progression and early relapse through mechanisms such as myeloid cell recruitment or TAM polarization (141). Another example is the colony-stimulating factor 1 (CSF1)/colony-stimulating factor 1 receptor (CSF-1R) axis, which is a key regulator of myeloid cell differentiation and chemotaxis, and which has been associated with BC progression and mortality (142). However, several treatments targeting these signaling pathways have been investigated in early phase clinical trials and have had disappointing results in the clinic to date, in part due to compensatory feedback mechanisms with no long-term benefit in solid tumors or metastatic cancers. Depletion of TAM could also be a valuable approach to facilitate the antitumor immune response in BC. For example, trabectidin (Yondelis) is effective at killing TAMs in addition to cancer cells (143). Therefore, its antitumor activity should be investigated in some BC subtypes with a high proportion of TAM, such as HR-positive BC (11), where TAM have been associated with worse survival (144). Remarkably, trabectidin has already shown a manageable safety profile and up to 56% of stable disease in several clinical trials with advanced or metastatic BC patients (145). In heavily pretreated metastatic HER2-positive or TNBC patients, partial response occurred in 12% of HER2-positive BC patients (146). In addition, zoledronic acid, which is commonly used in bone metastatic patients, showed extra-squeletal beneficial effects such as the prevention of distant relapse in BC patients after menopause (147), likely through TAMs depletion (148). One of the limitations of such myeloid immunotherapeutic strategies is the targeting of macrophages as a whole population, without taking into consideration the functional properties of subpopulations of macrophages. Indeed, some macrophage subtypes have for example been correlated with less advanced stages, less aggressive tumors (149) and better survival (150) in BC patients, while other subtypes have been suggested to be an independent prognostic marker for longer DFS and OS (151). In this context, another strategy aims at targeting a specific so-called “M2-like” subpopulation of macrophages or switching TAM polarization toward an antitumor phenotype, rather than depleting macrophages indiscriminately (152). Modulators of macrophage phenotype are tested in clinical trials in patients with solid tumors including BC patients, in combination with ICI (NCT02637531).

## Radiotherapeutics

Radiotherapeutics is based on a theranostic strategy that combines a whole-body non-invasive mapping of the cancer disease (through a targeted radioactive drug) and the delivery of a targeted therapy to the cancer cells (through a second radioactive drug). Although evidence is still limited, radiotherapeutics is emerging as a superior approach when compared to usual 18F-FDG PET-CT approach in detecting primary and metastatic BC disease. In addition, radiotherapeutics may help select patients who will benefit from therapy, and can become a new efficient targeted therapeutic option in metastatic setting. For example, the radiolabelling of trastuzumab with zirconium-89 or lutetium-177 has been suggested as specific radioimmunotherapy for HER2-positive BC patients (153, 154). Clinical trials assessing Her2 expression detection and anti-HER2 radionuclide therapy are currently ongoing in BC patients (NCT04674722)(NCT04467515). Prostate-specific membrane antigen (PSMA) is specifically expressed in tumor-associated vasculature of solid tumors such as BC, suggesting that it may be targeted as a new anti-angiogenic therapy. Progressive TNBC patients are currently being recruited to assess the concordance between lesions observed on Ga-PSMA PET-CT and 18F-FDG PET-CT and evaluate the feasibility of lutetium-177 PSMA therapy (NCT06059469). Another example is the Fibroblast Activation Protein (FAP)-targeted radionuclide therapy such as lutetium-177-FAPi which targets FAP-expressing Cancer-Associated Fibroblasts (CAFs), stroma cells from the TME endowed mainly with protumoral and immunosuppressive properties. Several studies suggested feasibility, safety, detection of metastases in specific areas (such as in the brain), and reduction of pain in metastatic BC (155, 156). Many other therapeutic isotopes and immunosuppressive targets within the TME might provide new radiotherapeutic and palliative tools for metastatic BC patients.

## Oncolytic viruses

Oncolytic viruses have been produced within the last decade. They are engineered to (or they preferentially) target tumor cells. Then, viruses replicate specifically in tumor cells, and stimulate antitumor immunity, ultimately killing tumor cells. This is mediated by the release of damage-associated molecular patterns and pathogen-associated molecular patterns, along with tumor-specific or tumor-associated antigens presentation. Furthermore, the oncolytic viruses induce immunogenic tumor cell death. Talimogene laherparepvec, a genetically modified herpes simplex oncolytic virus, has been approved by the FDA for the local treatment of metastatic melanoma with unresectable cutaneous lesions. In BC, many preclinical studies suggest antitumor effects of oncolytic virotherapy and its synergistic effects with chemotherapy.

The oncolytic herpes virus HF10 has been injected in BC patients with recurrent BC and suggested higher tumor-infiltrating CD8+ T cells, although the number of patients involved in the study was limited (157). Given the limited efficacy observed in single virotherapy to date, combination drug approaches including virotherapy are being further evaluated in BC patients. For example, a phase 2 randomized study of paclitaxel alone or in combination with oncolytic reovirus demonstrated, in 74 previously treated metastatic BC patients, a significantly longer OS when the combination treatment was administered (158). In another study, SD were observed in metastatic TNBC patients treated by virotherapy associated with low-dose cyclophosphamide (159). Oncolytic virotherapy is currently being investigated in advanced and metastatic BC patients in association with chemotherapy (NCT01656538)(NCT02630368), chemotherapy and ICI (NCT02630368)(NCT02977156) (NCT04215146). The sequence of the therapies over time might also be important in the efficacy of combinatorial therapeutic strategies. For example, preliminary oncolytic virotherapy has been suggested to sensitize BC to following chemo- or immunotherapy, since it induces a preexisting non-exhausted antitumor immunity (160). In this way, clinical trials are now recruiting BC patients for oncolytic virotherapy in association with neoadjuvant chemotherapy (NCT02779855)(NCT03564782), or with radiation therapy followed by pembrolizumab (NCT03004183). In addition, new immunotherapeutic strategies, such as oncolytic virotherapy coding for localized trastuzumab monoclonal antibody production (161), may provide synergistic antitumor effects. Non-replicating virus strategies have also been investigated. One strategy consists of the delivery of a gene that converts a drug into a cytotoxic drug. For example, a phase 1 trial used a local injection of a retrovirus encoding the human cytochrome P450 gene in BC metastatic cutaneous nodules in association with oral administration of the prodrug cyclophosphamide. The trial suggested safety and antitumor efficacy (162).

## Cytokine-based immunotherapy

Cytokines are major and pleiotropic regulators of the immune response. In the era of cancer immunotherapy, a renewed interest in the properties of cytokines has led to an increased number of clinical trials assessing their safety and efficacy. In BC, since cytokine-based immunotherapy has shown limited efficacy up to now, cytokines in association with other immunotherapies are currently being investigated to increase their efficacy. For example, it has been hypothesized that interferon-α (IFN-α), which upregulates tumor antigen presentation on tumor cells, might synergize with a cancer vaccine. Therefore, IFN-α has been administered in association with the CEA vaccine in thirty-three CEA-expressing cancer patients including BC. The administration of IFN-α induced a significantly increased OS compared to the vaccine alone (163). Interferon-γ (IFN-γ) is another cytokine which plays a crucial role in tumor cell cytotoxicity, and has also been suggested to be used as an adjuvant for immunotherapy. Hence, clinical trials are for example currently studying the association of IFN-γ with paclitaxel, trastuzumab and pertuzumab in HER-2-positive BC patients (NCT03112590), or with ICI in TNBC (NCT02614456). TGF-β is a cytokine which contributes to the immune suppression of the TME and has been associated with resistance to cancer immunotherapy. Its blockade during radiotherapy in metastatic BC patients has shown a higher median OS (164). Other major pro-inflammatory cytokines, such as IL-12 or IL-15, stimulate among others the production of IFN-γ from CD8+ T cells and induce the differentiation of CD4+ T cells into Th1. IL-12 or IFNγ, in combination with ICI and chemotherapy, may synergize efficiently. Indeed, these cytokines play key roles in the crosstalk between myeloid and lymphoid cells and in cellular cytotoxicity. This is currently under clinical investigation (NCT03567720)(NCT03112590). However, the severe IL-12-mediated toxicity restricted its use in clinical trials. IL-15 is currently investigated in association with ICI in patients with refractory cancers such as BC (NCT03388632). Finally, IL-2 plays a crucial role by stimulating, among others, the proliferation and the cytotoxicity of CD8+ T cells, but also the proliferation of regulatory T cells, which are major suppressors of the antitumor immune response. To overcome these limitations, engineered IL-2 and new inhibitors of regulatory T cell activity are under investigation. ICI have been suggested to inhibit regulatory T cell activity, and are therefore investigated in association with IL-2 in advanced cancers including BC (NCT05086692). The properties of cytokines make them a promising additional tool in cancer immunotherapy and will probably help facilitate the antitumor immune response, once they will be better understood and exploited.

## Immunometabolic targets

Accumulating evidence indicate a metabolic competition for consumption of glucose, amino acids and fatty acids between the tumor cells and the tumor-infiltrating immune cells. These molecules are essential for immune cell survival and activity, modulating in this way the antitumor immune response. This led to the concept of metabolic reprogramming. For example, enzymes such as arginase-1 (Arg-1) or indoleamine 2,3-dioxygenase (IDO-1), which catabolize arginine and tryptophan respectively, seem to be mainly involved in immunosuppressive pathways. Therefore, they have been targeted in BC preclinical models, demonstrating synergistic antitumor effects in association with chemotherapy (165, 166) and ICI (167). There are still a few immunometabolic targets investigated in BC clinical trials to date. A randomized clinical trial with HER2-negative metastatic BC patients failed to show improved PFS when IDO-1 inhibitor was added to chemotherapy (168). In contrast, IDO-1 inhibitor in association with p53-DC vaccine has been shown to increase the IFN ℽ-producing CD8 and the IL-2-producing CD4 T cell response in phase 1/2 study of metastatic BC patients, although it did not increase the objective response rate (169). Arginase inhibition, alone or in combination with ICI, is currently being evaluated in phase 1 study in advanced or metastatic cancer patients (NCT02903914). Accumulating evidence suggests that metabolic changes in adipose tissue are also associated with immunological dysregulations in BC (170). Fasting, or anti-hyperglycemic agents such as metformin, may modulate the antitumor immune response, improve the response to chemo- and immunotherapies, and reduce side effects (171). While fasting has been suggested to reduce the risk of BC recurrence (172), fasting (NCT05023967) and other metabolic health patterns (NCT05432856) are currently being clinically investigated. In addition, in most preclinical studies, hypoxia within the TME, and its associated VEGF induction, also contributes to tumor immune escape mechanisms and tumor progression. Moreover, hypoxia generally increases along with tumor progression, further promoting its deleterious effects (173). Furthermore, hypoxia increases extracellular levels of adenosine in the TME. Adenosine binds to its receptors on immune cells and further contributes to the establishment of an immunosuppressive TME (174). Rapidly proliferating malignant cells generate also high amount of lactate, a by-product of tumoral aerobic glycolysis. Lactate contributes to acidosis, stimulates angiogenesis, acts as cancer cell metabolic fuel, exerts deleterious effects on tumor-infiltrating immune cells and has been suggested to predict response to immunotherapy (175). Unfortunately, hypoxia-inducible factor 1-α (HIF-1α) and lactate metabolism inhibitors have mainly been investigated at a basic research level up to now or have only occasionally been clinically assessed. Adenosine has emerged as a key negative regulator of antitumor immunity through the CD39-CD73-A2AR pathway (176). Several inhibitors of this pathway are currently being evaluated in early phase clinical trials alone or in combination with ICI or chemotherapy in patients with advanced solid tumors including BC patients (NCT02740985)(NCT02503774)(NCT02754141), with trastuzumab (NCT05143970) or in combination with radiotherapy (NCT03875573). Nevertheless, metabolic reprogramming is emerging and require further preclinical and clinical investigations before leading to safe and efficient immunotherapeutic adjuvants for BC immunotherapies.

## Discussion

It is well established that the TME plays a crucial role in cancer outcomes and response to therapy. Indeed, it provides a supportive but also active protumoral and immunosuppressive framework for tumor progression and dissemination. On the other hand, evidence demonstrating how to harness the immune system in favor of strong antitumor immunity is overflowing. To improve research and development in BC immunotherapy, clinical trials must be critically designed, considering both molecular and cellular mechanisms at play and its clinical pitfalls.

The next generation of cancer immunotherapy will probably involve combination immunotherapies. Indeed, the current approach of cancer immunotherapy mainly focuses on the T-cell compartment, allowing one to speculate that additional complementary immunotherapeutic strategies targeting different immune compartments could lead to synergistic therapeutic approaches. More specifically, numerous clinical trials assess immunotherapeutic combinations that mechanistically regulate redundant pathways (such as the combination of two different ICI for example). Instead, targeting different complementary pathways could trigger a stronger antitumor immune response. Specifically, the combination of myeloid and lymphoid ‘‘immune checkpoints’’ should be investigated further. Lymphoid immune checkpoints on T cells are widely studied. In contrast, myeloid cells (such as macrophages and dendritic cells) and their potential associated therapeutic targets are clinically less well-known. However, the myeloid cells are still key modulators of the adaptive immune system. Indeed, they can cooperate with TILs to develop a strong and long-lasting antitumor immune response together. Unfortunately, the tumor modulates its TME. Instead of promoting an inflammatory response, many preclinical studies demonstrated that most tumor-associated myeloid cells strongly suppress TILs. Therefore, there has been an explosive growth of clinical trials targeting tumor-infiltrating myeloid cells worldwide, most of them still at an early phase (177, 178). However, targeting only the lymphoid or the myeloid compartment of the TME may not adequately restore the antitumor immune response. We believe that restoring a positive crosstalk between tumor-infiltrating myeloid and lymphoid immune cells may optimize BC immunotherapy.

Distinct macrophages and dendritic cells have been suggested to predict the response to ICI immunotherapy in human BC (179, 180) and other solid tumors (181, 182). An antitumor vaccine has demonstrated long-term tumor control in a HER2-positive BC model but only when combined with anti-PD-1 treatment. This has led to the investigation of this therapeutic combination in a phase 2 clinical trial (NCT03632941). Furthermore, a first-in-human phase 1 trial supports further clinical investigation of evorpacept, a protein that promotes tumor phagocytosis by dendritic cells and macrophages, combined with pembrolizumab, in patients with solid tumors (139). Lastly, eganelisib, a potential first-in-class tumor macrophage-targeting agent (NCT02637531), is already showing PFS benefit in metastatic TNBC patients in addition to atezolizumab and nab-paclitaxel in an ongoing phase 2 MARIO-3 trial (NCT03961698).

Moreover, compensatory mechanisms are very often at play in the TME, further suggesting the need for combination immunotherapies. For example, several studies suggested an increased immunosuppressive microenvironment after CAR T cell therapy (102). CAR T-cells infusion without targeting the TME in parallel, which suppresses TILs, might seem senseless. New complementary strategies aiming at improving T-cell trafficking into/proliferation within the TME, such as myeloid cell depletion, may improve CAR T-cell efficacy. In addition, tumor PD-L1 upregulation occurs in response to IFNγ release by effector immune cells, leading to subsequent immune suppression, a process known as adaptive immune resistance, where tumor cells protect themselves from immune attack (108). Therefore, the addition of ICI to other types of immunotherapies, such as cancer vaccines for example, may restrain induced immunosuppressive feedback. Moreover, combinations of conventional therapies and immunotherapies should be investigated. Indeed, although radiotherapy and chemotherapy can result in immunogenic cell death (183), they can also limit their own therapeutic effects. For example, conventional cytotoxic drugs and vascular-targeting agents induce tumor cells to produce macrophage recruitment factors (136), while macrophages can also be recruited and polarized during radiotherapy treatment (184). This promotes tumor tissue repair and early tumor recurrence, which might be thwarted by myeloid-targeting immunotherapeutic strategies. Other myeloid and lymphoid-based treatment combinations involving, in addition to an ICI, a CD40 agonist (NCT03424005) or an antitumor vaccine (NCT03632941), may synergize by activating dendritic cells. An additional rational combination might be the concomitant blockade of the CD47/SIRPα axis (or other anti-phagocytic signal blockade) with trastuzumab, which might provide a synergistic phagocytic antitumor activity. In accordance with this hypothesis, CD47 gene expression has been found to limit the therapeutic activity of trastuzumab in HER2-positive BC patients (15). In addition, some treatments such as corticosteroids, mainly used for symptomatic purposes in oncology, should be administered carefully, since their impact on the antitumor immune response is still not well understood (185). The sequence of immunotherapies over time might also impact their efficacy. Indeed, the TME involves a complex dynamic network of immune cells interacting with each other, displaying changes in their activation state over time, and in this way affecting tumor progression and response to therapy. This concept is underestimated in trials evaluating heavily pretreated metastatic BC patients and excluding earlier settings. Furthermore, the dose and schedule of therapies play an important role in their activities. For example, Gonadotropin-Releasing Hormone (GnRH) agonists achieve castration because of continuous pituitary stimulation in contrast to the physiologic pulsatile fashion. Another example is cyclophosphamide, which has cytotoxic and immunosuppressive effects at high dosage, but displays immunostimulatory and antiangiogenic effects at a daily lower dose. New trial designs involving metronomic chemotherapies in association with ICI should be done in search of the optimal antitumor activity (NCT03971045). In a near future, new technologies such as machine learning will probably be a tool of an inestimable value to help us better understand immune cell subpopulation activities and interactions under therapies, and suggest efficient combinations of therapies.

Predictive biomarkers of response to immunotherapy are sorely lacking today and must be developed. Indeed, PD-1 and PD-L1 expression as biomarkers are not reliable enough, probably because the response to therapy is much more complex than that of those sole molecules. While TILs are considered in clinical practice as a promise of good prognosis (186, 187) and response to therapy, they consist of many subpopulations of lymphocytes exerting various and sometimes opposite immune functions (188). For example, cytotoxic CD8+ T cells and CD4+ Th1 T cells are associated with BC survival. On the other hand, potent immunosuppressive TILs such as regulatory T cells are associated with poor BC clinical outcome. As seen previously, different tumor-infiltrating myeloid cells can also exert critical opposite functions around the tumor cells and in metastatic niches. Based on the characterization of various tumor-infiltrating immune cell subpopulations and their correlations with clinical outcomes, a cytotoxic/regulatory immunogenic ratio might be conceived and used as a new predictive biomarker of response to therapy in future biopsies.

To conclude, immunotherapy using ICI has shown unprecedented and long-term efficacy in the treatment of cancer patients with various advanced solid tumors in the last decade, and is currently used in many oncology treatments and clinical trials (189). It has become one of the pillars of cancer treatment, but its success is currently still the visible tip of the iceberg. Indeed, countless efforts have been made to develop cancer immunotherapies and multiple new promising therapies are likely to emerge. Meanwhile, many challenges are still to be overcome, and the next decade will see the fruitfulness of BC immunotherapy investigations, including appropriate synergistic combinations with standards of care.

## Author contributions

EA: Writing – original draft, Writing – review & editing. MS: Writing – review & editing. AS: Writing – review & editing.
